# Integrated biology approach reveals molecular and pathological interactions among Alzheimer’s Aβ42, Tau, TREM2, and TYROBP in *Drosophila* models

**DOI:** 10.1186/s13073-018-0530-9

**Published:** 2018-03-29

**Authors:** Michiko Sekiya, Minghui Wang, Naoki Fujisaki, Yasufumi Sakakibara, Xiuming Quan, Michelle E. Ehrlich, Philip L. De Jager, David A. Bennett, Eric E. Schadt, Sam Gandy, Kanae Ando, Bin Zhang, Koichi M. Iijima

**Affiliations:** 1 Department of Alzheimer’s Disease Research, National Center for Geriatrics and Gerontology, 7-430 Morioka-cho, Obu, Aichi 474-8511 Japan; 2 Department of Genetics & Genomic Sciences, Icahn School of Medicine at Mount Sinai, 1470 Madison Avenue, Room 8-111, Box 1498, New York, NY 10029 USA; 3 Icahn Institute of Genomics and Multiscale Biology, Icahn School of Medicine at Mount Sinai, One Gustave L. Levy Place, New York, NY USA; 4 Department of Experimental Gerontology, Graduate School of Pharmaceutical Sciences, Nagoya City University, 3-1 Tanabe-dori, Mizuho-ku, Nagoya, Japan; 5 Department of Neurology, Alzheimer’s Disease Research Center, Icahn School of Medicine at Mount Sinai, New York, NY USA; 6 Department of Pediatrics, Icahn School of Medicine at Mount Sinai, New York, NY USA; 7 Center for translational & Computational Neuroimmunology, Department of Neurology, The Neurological Institute of New York, Columbia University Medical Center, New York, NY USA; 8 Broad Institute, Cambridge, MA USA; 9 Rush Alzheimer’s Disease Research Center and Department of Neurology, Rush University Medical Center, 1750 W. Congress Parkway, Chicago, IL 60612 USA; 10 Department of Psychiatry and Alzheimer’s Disease Research Center, Icahn School of Medicine at Mount Sinai, New York, NY USA; 11 Center for NFL Neurological Care, Department of Neurology, New York, NY USA; 12 James J. Peters VA Medical Center, 130 West Kingsbridge Road, New York, NY USA; 13 Department of Biological Sciences, Graduate School of Science and Engineering, Tokyo Metropolitan University, Tokyo, Japan; 14 Ronald M. Loeb Center for Alzheimer’s Disease, Icahn School of Medicine at Mount Sinai, One Gustave L Levy Place, New York, NY USA

**Keywords:** Alzheimer’s disease, Amyloid-β (Aβ) peptides, Microtubule-associated protein tau, *TYROBP* (tyrosine kinase binding protein), *TREM2* (triggering receptor expressed on myeloid cells 2), Differential expression, Gene co-expression network, Gene module, Synaptophagy, Immune function, Neurodegeneration

## Abstract

**Background:**

Cerebral amyloidosis, neuroinflammation, and tauopathy are key features of Alzheimer’s disease (AD), but interactions among these features remain poorly understood. Our previous multiscale molecular network models of AD revealed *TYROBP* as a key driver of an immune- and microglia-specific network that was robustly associated with AD pathophysiology. Recent genetic studies of AD further identified pathogenic mutations in both *TREM2* and *TYROBP*.

**Methods:**

In this study, we systematically examined molecular and pathological interactions among Aβ, tau, TREM2, and TYROBP by integrating signatures from transgenic *Drosophila* models of AD and transcriptome-wide gene co-expression networks from two human AD cohorts.

**Results:**

Glial expression of TREM2/TYROBP exacerbated tau-mediated neurodegeneration and synergistically affected pathways underlying late-onset AD pathology, while neuronal Aβ42 and glial TREM2/TYROBP synergistically altered expression of the genes in synaptic function and immune modules in AD.

**Conclusions:**

The comprehensive pathological and molecular data generated through this study strongly validate the causal role of *TREM2/TYROBP* in driving molecular networks in AD and AD-related phenotypes in flies.

**Electronic supplementary material:**

The online version of this article (10.1186/s13073-018-0530-9) contains supplementary material, which is available to authorized users.

## Background

Alzheimer’s disease (AD) is the leading cause of neurodegeneration and dementia. At the level of neuropathology, AD is characterized by aggregation and accumulation of two proteins, β-amyloid peptides (Aβ) and the microtubule-associated protein tau [1]. It is accompanied by the activation of multiple neuroinflammatory pathways [2]. Lines of evidence from laboratories and clinics worldwide support the concept that accumulation of Aβ peptides can be an initiating factor and can lie upstream of tau to drive synaptic dysfunction, neuron death and cognitive impairment [3–7].

A new model was developed to account for the fact that up to one-third of patients with clinically diagnosed AD have no evidence of amyloidosis on brain amyloid imaging [8]. Alternatively, some older individuals with neuropathological AD were asymptomatic during their lifetime [9]. These clinicopathological studies indicate that disease progression is a complex process resulting from the interplay of a number of genetic and environmental factors, some of which modulate accumulation of neuropathology while others modulate synaptic and neuronal resilience [10]. System-level analyses of large datasets from patients have emerged as powerful tools for understanding complex diseases such as AD. Gene expression datasets, along with genomic and clinical information from multiple studies, continue to accumulate and data interpretation is becoming a difficult challenge in these “omics” approaches.

Gene regulatory network analysis is a powerful tool in identifying gene modules pathologically related to human complex diseases including AD [11, 12]. We employed an integrative multiscale network analysis approach to identify key molecular interactions of cellular pathways and causal regulators underlying pathological changes in AD. This approach identified *TYROBP* (tyrosine kinase binding protein, also known as *DAP12*), the intracellular adaptor of *TREM2* (triggering receptor expressed on myeloid cells 2), as a key driver of immune- and microglia-specific networks that are associated with LOAD pathology [11, 13]. Genome-wide association studies (GWAS) revealed that *TREM2*, a *TYROBP*-binding protein, is a risk gene for late-onset sporadic AD [14–16]. More recently, *TYROBP*-coding sequence genetic variants were found to contribute to an increased risk of early-onset AD [17]. Moreover, TREM2/TYROBP signaling is upregulated by plaque-associated myeloid cells in AD brains and in APP transgenic mice [18–20]. An ectodomain of TREM2 is cleaved and released into the extracellular space as a soluble form (sTREM2) and sTREM2 levels in CSF are elevated in the early symptomatic phase of AD [21–23]. Interestingly, this cleavage of TREM2 is reduced by pathogenic mutations for AD [24, 25]. These reports underscore the role of *TREM2/TYROBP* in AD pathogenesis.

*TREM2* encodes a receptor expressed exclusively in the immune cells in the brain [26, 27]. Studies with *TREM2*-deficient AD model mice suggest that TREM2 may influence phagocytosis of Aβ-lipid complexes as well as microglial survival [28] and metabolic fitness [29]. Microglia forms a barrier to restrict Aβ plaque growth and diffusion of soluble Aβ oligomers [30], thereby ameliorating tau pathology in AD mouse models [31–33]. *TREM2* deficiency or the AD-associated R47H mutation in *TREM2* significantly reduced accumulation of microglia around Aβ plaques [28, 34]. A recent study also shows that some effects of *TREM2* on Aβ pathology may be disease-stage-dependent [33].

By contrast, activation of microglia can play not only beneficial but also detrimental roles in plaque-related neuropathology. Microglia in adult brains engulf spines and other synaptic processes; exposure to Aβ may inappropriately activate this process to mediate synapse loss [35]. This “synaptophagy” involves complement and CR3, which, like TREM2, can provide an ectodomain protein that interacts with TYROBP [36]. Intriguingly, *TYROBP* deficiency in APPswe/PS1dE9 mice reduces plaque-associated microglia with improved electrophysiological and learning behavior effects [37]. In addition, TREM2 overexpression failed to improve neuropathology and cognitive impairment in aged APPswe/PS1dE9 mice [38]. More recent studies demonstrate that TREM2 pathway promotes the transition from homeostatic to disease-associated microglia in brains of AD model mice [39, 40].

These reports are consistent with a model wherein TREM2/TYROBP signaling is activated as a protective response against Aβ pathology; however, sustained TREM2/TYROBP activation may ultimately aggravate inflammatory and synapse-related pathologies, thereby driving AD progression. Thus, elucidation of the molecular basis for various interrelationships involving Aβ, TREM2/TYROBP, and tau may fill some gaps in our understanding of AD pathogenesis.

In this study, we aimed to decipher molecular interactions among Aβ, TREM2/TYROBP, and tau by integrating gene expression signatures associated with TREM2/TYROBP from AD *Drosophila* models and transcriptome-wide gene co-expression networks from two human AD cohorts including Harvard Brain Tissue Resource Center (HBTRC) [11], and the Religious Orders Study and the Rush Memory and Aging Project (ROSMAP) [41, 42]. The impact of an AD-associated TREM2 R47H variant on these molecular interactions was also analyzed. Our data demonstrate that co-expression of neuronal Aβ42 with glial TREM2^R47H^/TYROBP led to synergistic downregulation of genes associated with synaptic function modules in fly brains. Moreover, glial expression of both TREM2^WT^/TYROBP and TREM2^R47H^/TYROBP exacerbated tau toxicity and synergistically affected the pathways implicated in AD-related neurodegeneration. Thus, gene regulatory networks highlighted by this unbiased, cross species analysis appeared to recapitulate some key features of AD progression and support a key driver role for *TREM2/TYROBP* in AD pathogenesis.

## Methods

### Drosophila genetics

Flies were maintained in standard cornmeal media at 25 °C. Complementary DNA (cDNA) encoding the full length of *TREM2* (NM_018965, RC221132) and *TYROBP* (NM_198125, RC203771) with Myc-DDK tag were obtained from OriGene Technologies, Inc. These constructs were subcloned into a pJFRC19-13XLexAop2 vector (Addgene #26224). *TREM2R47H* mutation was introduced by using site-directed mutagenesis kit (Takara Bio Inc.). Transgenic flies were generated by PhiC31 integrase-mediated transgenesis systems (Best Gene Inc.). Transgenic fly lines carrying UAS-Aβ42 and UAS-tau were previously described [43–45]. Repo-LexA (#67096), Elav-GAL4 (#458), GMR-GAL4 (#1104), UAS-para RNAi (#31626), and UAS-mcherry RNAi (#35785) were obtained from the Bloomington Stock Center. For RNA sequencing (RNA-seq), around seven-day-old male flies were used. All experiments were performed using age-matched male flies.

#### Western blotting

Fly heads for each genotype were homogenized in appropriate buffer and subjected to western blotting or co-immunoprecipitation. Details for western blotting and sequential extractions of Aβ42 were performed as previously described [44]. Anti-FLAG (Sigma-Aldrich), anti-TREM2 (Cell signaling), anti-tau (Millipore), anti-non phospho tau (Merck Millipore), anti-pThr231 tau (Thermo Fisher Scientific), anti-pSer262 tau (Abcam), and anti-tubulin (Sigma-Aldrich) for western blotting were purchased.

#### Histological analysis

Heads of male or female flies were fixed in 4% paraformaldehyde for 24 h at 4 °C and embedded in paraffin. Serial sections (6-μm thickness) through the entire heads were prepared, stained with hematoxylin and eosin (Sigma-Aldrich), and examined by bright-field microscopy. Images of the sections were captured with AxioCam 105 color (Carl Zeiss); the vacuole area was measured using Image J (NIH).

#### Climbing assay

Approximately 25 flies were placed in an empty plastic vial. The vial was then gently tapped to knock all of the flies to the bottom. The numbers of flies in the top, middle, or bottom thirds of the vial were scored after 10 s. The percentages of flies that stayed at the bottom were subjected to statistical analyses. Experiments were repeated more than three times and a representative result was shown.

#### Courtship-conditioning assay

Courtship-conditioning assay was performed using the method by Ishimoto *et al*. [46, 47]. Unreceptive, mated-females were prepared as “trainers” one day before the conditioning. For training, a three- to five-day-old virgin male was placed with a trainer female in the courtship chamber (15 mm in diameter × 5 mm in depth) for 1 h. Trained males and non-trained naïve males were tested with freeze-killed virgin females as a courtship target in the courtship chamber 1 h after training. The courtship index (CI) was defined as the proportion of time spent in courtship behavior during 10 min observation period. We used more than 60 flies for each genotype. CIs for conditioned males and naïve controls were analyzed by Mann–Whitney *U* test. To compare the memory performances of each genotype, experimental data are presented as the performance index (PI), which was calculated using the following formula. PI = 100 × (CI ^average for naïve^ – CI ^conditioned^)/CI ^average for naïve^, after CIs were subjected to arcsine square root transformation to approximate normal distributions.

#### Reverse transcription polymerase chain reaction (RT-PCR) and quantitative reverse transcription polymerase chain reaction (qRT-PCR)

RNA extraction was described below (RNA-seq and analyses). Total RNA was reverse-transcribed using PrimeScript RT-PCR kit (TaKaRa Bio) and the resulting cDNA was used as a template for PCR (Veriti, Applied Biosystems). PCR products were analyzed by 1% agarose gel.

qRT-PCR was performed using PowerSYBR (Thermo Fisher Scientific) on a CFX96 real-time PCR detection system (Bio-Rad Laboratories). The average threshold cycle value (CT) was calculated from at least three replicates per sample. Expression of genes of interest was standardized relative to GAPDH1. Relative expression values were determined by the ΔΔCT method. Primers were designed using Primer-Blast (NIH) or FlyPrimerBank [48] as described in Additional file 1: Table S11.

#### RNA sequencing and analyses

More than 100 flies for each genotype were collected and frozen. Heads were mechanically isolated and total RNA was extracted using TRIzol Reagent (Invitrogen, Thermo Fisher Scientific) according to the manufacturer’s protocol with an additional centrifugation step (16,000 × g for 10 min) to remove cuticle membranes before the addition of chloroform. Total RNA was purified using phenol-chloroform reagents after treatment with DNAaseI.

Preparation of samples for RNA-seq analysis was performed using the TruSeq RNA Sample Preparation Kit v2 (Illumina). Briefly, ribosomal RNA was depleted from total RNA using the Ribo-Zero rRNA Removal Kit (Human/Mouse/Rat) (Illumina) to enrich for coding RNA and long non-coding RNA. The cDNA was synthesized using random hexamers, end-repaired, and ligated with appropriate adaptors for sequencing. The library then underwent size selection and purification using AMPure XP beads (Beckman Coulter). The appropriate Illumina recommended 6-bp bar-code bases are introduced at one end of the adaptors during PCR amplification step. The size and concentration of the RNA-seq libraries were measured by Bioanalyzer (Agilent) and Qubit fluorometry (Life Technologies, Thermo Fisher Scientific) before loading onto the sequencer. The Ribo-Zero libraries were sequenced on the Illumina HiSeq 2500 System with 100 nucleotide single-end reads, according to the standard manufacturer’s protocol (Illumina).

Single-ended RNA-seq data were generated with the Illumina HiSeq 2500 platform following the Illumina protocol. The raw sequencing reads were aligned to fly genome BDGP6 using star aligner (version 2.5.0b). Following read alignment, featureCounts [49] was used to quantify the gene expression at the gene level based on Ensembl gene model. Genes with at least 5 reads in at least one sample were considered expressed and hence retained for further analysis, otherwise removed. The gene level read counts data were normalized using trimmed mean of M-values normalization (TMM) method [50] to adjust for sequencing library size difference.

Differential gene expression between different genotypes was predicted by linear model analysis using Bioconductor package LIMMA [51]. RNA integrity number (RIN) score was incorporated as a covariate in the linear model to control for sample quality. To adjust for multiple tests, false discovery rate (FDR) of the differential expression test was estimated using the Benjamini–Hochberg (BH) method [52]. Genes with FDR < 0.05 and log2 fold change > 1 or < − 1 were considered significant.

#### Functional enrichment analysis (FEA)

For FEA of differential expression gene signatures, the gene ontology (GO) annotations were obtained from the flyBase database. Then the enrichment analysis was carried out using Fisher’s exact test (FET), assuming the genes in different sets were identically independently sampled from the genome-wide genes profiled. The BH approach was employed to constrain the FDR.

#### ROSMAP AD cohort network analysis

We utilized large-scale RNA-seq data of the ROSMAP AD cohort to build a gene co-expression network to capture the coordinated regulation of gene expression traits in brain samples. This dataset profiled gene expression of postmortem brain samples from two longitudinal studies of aging and AD [41, 42]. In both studies, participants enroll without dementia and agree to annual clinical evaluation and organ donation at death. As a result, most decedents are old, without dementia and few participants reach end stage dementia before death. They differ from the types of cases obtained in tertiary care clinics [53]. In the ROSMAP dataset, there were 1059 samples including 362 AD cases and 697 controls. We downloaded preprocessed RNA-seq FPKM gene expression abundance data, SNP genotype data, and DNA methylation data from Synapse (10.7303/syn3219045). We downloaded preprocessed RNA-seq FPKM gene expression abundance data from Synapse (10.7303/syn3388564). Genes with at least 1 FPKM in at least 10% of the samples were selected and then the data were corrected for confounding factors including batch, PMI, sex, and RIN score. Co-expression network was constructed by using R package WINA [12], which implements a computationally optimized procedure for weighted gene co-expression network analysis (WGCNA) [54]. In WINA analysis, we used power β = 6 with other parameters set by default. Thirty-five modules (i.e. clusters of gene showing highly correlated expression profiles across samples) were identified, which were annotated by the mostly enriched gene ontology/canonical pathway term. The modules were rank sorted in relation to AD pathology by multiple sorting features computed from the ROSMAP data, including module-trait correlations and enrichment for AD-related disease gene signatures including DEGs and trait-correlated genes (TCGs) regarding neuropathological/clinical traits such as Braak staging, cognitive score, CERAD neuropathological category, and NIA-Reagan score.

Bayesian causal network was constructed by integrating genome-wide gene expression, SNP genotype, DNA methylation, and known transcription factor (TF)-target relationships. Briefly, we first computed expression quantitative trait loci (eQTLs) and then employed a formal statistical causal inference test (CIT) [55] to infer the causal probability between gene pairs associated with the same eQTL. With a similar strategy, we also computed causal probability of gene pairs mediated or regulated by a DNA methylation site. The causal relationships inferred were combined with TF-target relationships, and together they were subsequently used as priors for building a causal network through a Monte Carlo Markov Chain (MCMC) simulation-based procedure [56].

#### Statistical analysis for biological assay

All results were expressed as mean ± SEM. Unpaired Student’s *t*-test (Prism7, GraphPad Software Inc.) was used to determine statistical significance as indicated in the figure legends. * indicates *p* < 0.05, ** indicates *p* < 0.01 and *** indicates *p* < 0.001 for biological assays throughout the manuscript.

#### Data availability

RNA-seq raw data have been deposited in the Gene Expression Omnibus (GEO) database under accession number GSE99012.

## Results

The hypothesis underlying this work was that TREM2/TYROBP plays a causal role in driving molecular networks in AD [11]. To test this hypothesis, we used *Drosophila* to identify molecular interactions between neuronal expression of Aβ42 or tau and glial expression of TREM2/TYROBP.

**Fig. 1:**
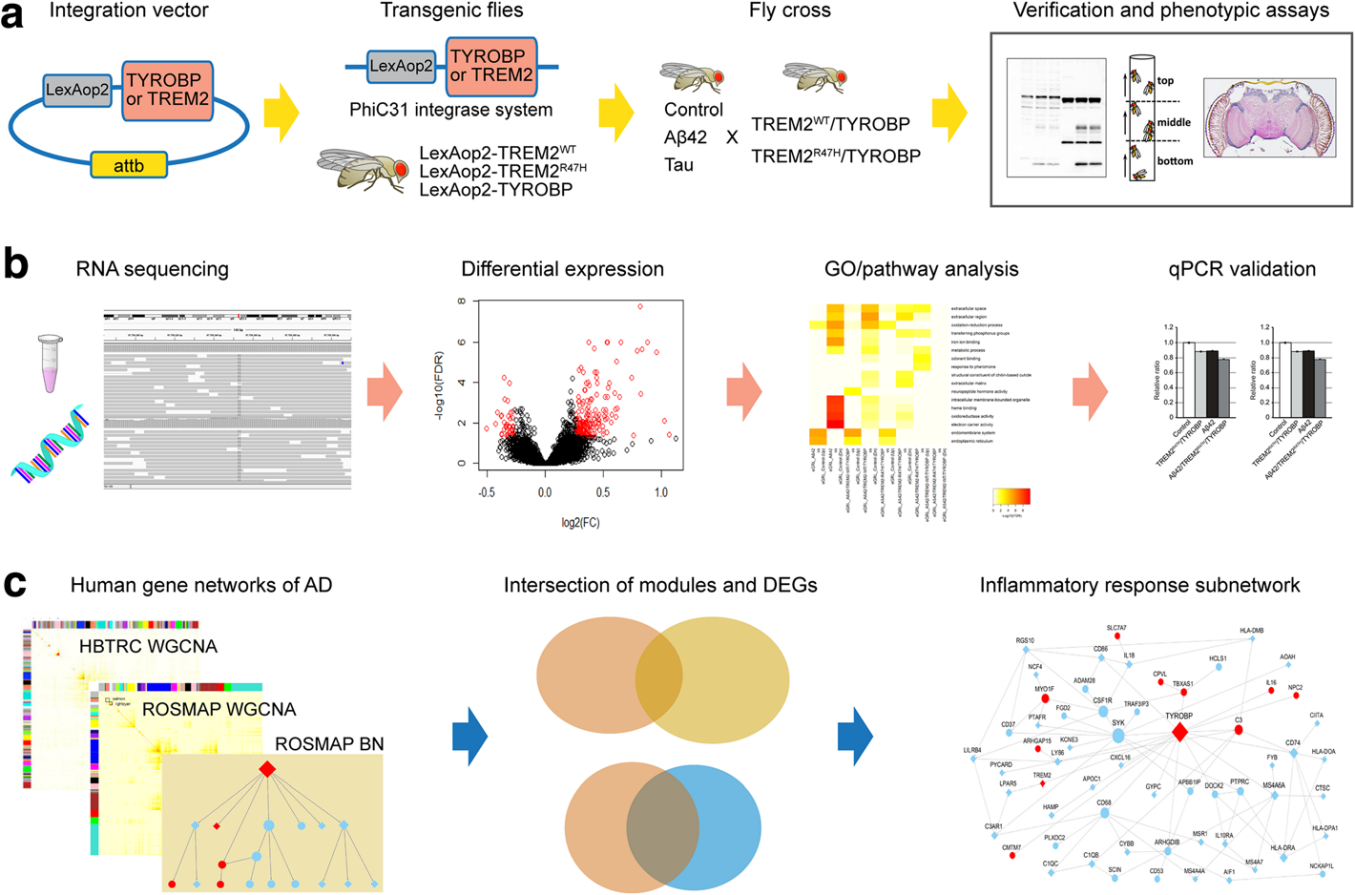
Overview of the present study design and establishment of transgenic flies co-expressing TREM2 with TYROBP. **a** Transgenic flies expressing or co-expressing human TREM2/TYROBP in glial cells, Aβ42 in neurons, or tau in the retina were developed. These fly models were characterized for several phenotypic changes. **b** RNA from control and transgenic fly heads were profiled by RNA-seq, to identify differentially expressed genes (DEGs). The gene ontology (GO) and pathway terms enriched for DEGs were identified, and several DEGs were validated by qPCR. **c** Two WGCNA (Weighted Gene Co-expression Network Analysis) co-expression networks and a Bayesian regulatory network (BN) were collected from gene expression datasets of two human AD cohorts (HBTRC and ROSMAP). Human orthologs of the fly DEGs were projected onto gene co-expression networks of human AD datasets to explore the relevance of these gene signatures to AD pathogenesis from a network prospective

Figure 1 shows an overview of the design and data analysis in the present paper. Briefly, we developed transgenic fly models expressing or co-expressing human TREM2^WT^/TYROBP or TREM2^R47H^/TYROBP in glial cells, Aβ42 in neurons, or tau in the retina. These transgenic fly models were characterized for several phenotypic changes including Aβ42 accumulation, the status of tau phosphorylation levels, behavioral deficits, and neurodegeneration. Then transcriptome-wide gene expression in control and transgenic fly heads were profiled by RNA-seq to identify differentially expressed gene (DEG) signatures between different genotype groups. The GO and pathway terms enriched in the DEG signatures were identified. Several DEGs were validated by qPCR. Lastly, human orthologs of the fly DEG signatures were projected onto gene networks from human AD datasets to explore the relevance of these gene signatures to AD pathogenesis from a network prospective. Gene regulatory relationship was characterized for a highlighted inflammatory response subnetwork using Bayesian network analysis.

### Establishment of transgenic flies co-expressing TREM2 (TREM2^WT^) or TREM2 with pathogenic R47H variant (TREM2^R47H^) with TYROBP in glial cells

**Fig. 2:**
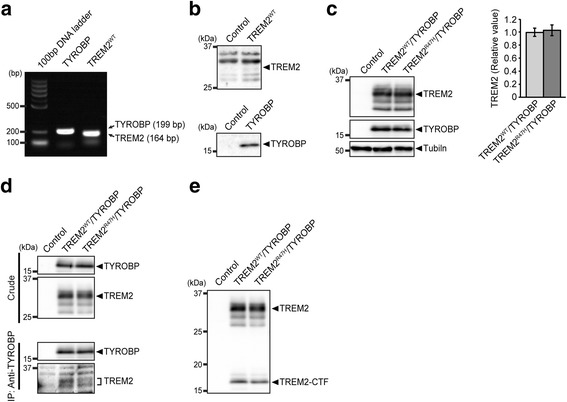
Establishment of transgenic flies co-expressing TREM2^WT^ or TREM2^R47H^ with TYROBP in glial cells. **a** mRNA expression of TREM2 or TYROBP driven by a pan-glial driver Repo-LexA was confirmed by RT-PCR analysis. **b** Western blotting of fly head expressing TREM2^WT^ or TYROBP driven by Repo-LexA. TREM2 and TYROBP were tagged with Myc-DDK (FLAG). Membranes were probed with anti-FLAG antibody. Control; Repo-LexA driver alone. **c** Western blotting of fly head co-expressing TREM2 with TYROBP driven by Repo-LexA. Membranes were probed with anti-TREM2 or anti-TYROBP antibody. Tubulin was used as a loading control. No significant difference. (Student’s *t*-test). **d** TYROBP co-immunoprecipitated with TREM2. Fly head lysates were subjected to immunoprecipitation with anti-TYROBP antibody, followed by western blotting with anti-TYROBP or anti-TREM2 antibody. *Top:* Western blotting of crude lysate. *Bottom:* Immunoprecipitate with anti-TYROBP antibody was subjected to western blotting. **e** Western blotting of fly head lysate co-expressing TREM2 and TYROBP by anti-TREM2 antibody detected a full length of TREM2 as well as the C-terminal fragment of TREM2 (TREM2-CTF). Genotypes of flies are described in Additional file 2: Table S1

In order to co-express human TREM2 and TYROBP in fly glial cells, we generated transgenic flies carrying wild-type (WT) human TREM2 (TREM2^WT^), TREM2 with AD-related R47H variant (TREM2^R47H^), or TYROBP under the control of a tissue-specific LexA operator [57]. Expression of each transgene was driven by a pan-glial driver Repo-LexA, and their messenger RNA (mRNA) expression was confirmed by RT-PCR analysis (Fig. 2a).

We found that, while the expression of TYROBP proteins was readily detectable by western blotting, TREM2 protein levels were undetectable, raising the possibility that ectopically expressed human TREM2 proteins may be unstable in fly glial cells perhaps because a binding partner that is required to stabilize TREM2 protein was absent (Fig. 2b). Indeed, when TREM2 and TYROBP transgenes were combined and co-expressed in fly glial cells, TREM2 proteins became readily detectable (Fig. 2c). A prior report showed that R47H mutation reduced the stability of TREM2 proteins [58]. We compared protein levels of TREM2^WT^ and TREM2^R47H^ in fly brains and found no significant difference between them (Fig. 2c). When TYROBP was immunoprecipitated from the lysate of bigenic fly brains co-expressing TYROBP and TREM2, TREM2 proteins (both TREM2^WT^ and TREM2^R47H^) were also precipitated, indicating that TREM2 and TYROBP proteins interact and stabilize each other in fly glial cells (Fig. 2d).

In mammalian cells, TREM2 is cleaved by α-secretase, which results in production of N- and C-terminal fragments of TREM2 [59]. The N-terminal fragments of TREM2 are secreted (sTREM2) and promote inflammatory responses [60], while C-terminal fragments of TREM2 are further processed by γ-secretase [59]. Western blotting using an anti-TREM2 antibody detected the C-terminal fragment of both TREM2^WT^ and TREM2^R47H^ (Fig. 2e), suggesting that TREM2 is processed and that sTREM2 is produced in fly glial cells in a manner similar to that observed in mammalian cells.

### FEA revealed that significant overlap between molecular pathways affected by neuronal expression of Aβ42 and those affected by glial expression of TREM2^WT^/TYROBP in fly brains

**Fig. 3:**
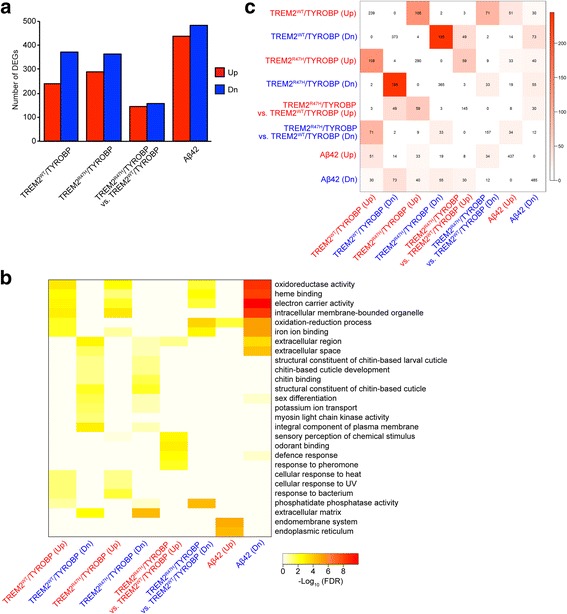
Molecular pathways affected by neuronal expression of Aβ42 overlap with those affected by glial TREM2/TYROBP. **a** Number of DEGs. The numbers of upregulated genes are indicated in *red* and the numbers of downregulated genes are in *blue*. **b** *Heatmap* showing the top functional pathways enriched in the DEGs identified in (**a**). The heatmap color intensity denotes the statistical significance of the enrichment (FDR at minus log 10 scale). **c** Overlaps among DEGs identified in (**a**). The number in each cell indicates the number of common DEGs between row and column variables, with color intensity indicating the FDR adjusted *p* value at minus log 10 scale. Genotypes of flies are described in Additional file 2: Table S1

To gain insights into the effects of glial expression of TREM2/TYROBP in the fly brains at the molecular level, we generated RNA-seq data from the brain samples in control flies (control) and flies with co-expression of TREM2^WT^ and TYROBP (TREM2^WT^/TYROBP). Differentially expressed genes between control and TREM2/TYROBP flies were identified using two criteria including fold change > 1.2 and a FDR < 0.05 in an analysis using linear models implemented using the R package LIMMA [61]. Expression of TREM2^WT^/TYROBP resulted in upregulation of 239 genes and downregulation of 373 genes (Fig. 3a and Additional file 2: Table S1).

Since TREM2/TYROBP signaling is known to promote survival of microglial cells [28], we evaluated whether ectopic expression of TREM2 and TYROBP induced any structural changes in the fly brain and/or significantly altered the number of glial cells or neurons. No significant alteration in the size or gross morphology of brain structures was observed in TREM2^WT^/TYROBP bigenic flies (Additional file 1: Figure S1). In addition, immunostaining of fly brains against a glial marker protein, Repo, or a neuronal marker, Elav, revealed that the numbers of glial cells or neurons were not significantly different between control and TREM2^WT^/TYROBP bigenic fly brains (Additional file 1: Figure S1). These results suggest that gene expression changes induced by ectopic expression of TREM2^WT^/TYROBP are not due to either structural defects or altered number of neurons or glial cells in the fly brain.

To identify the biological pathways that are affected by glial expression of TREM2^WT^/TYROBP, we performed functional enrichment analysis (FEA) for the DEG signatures using GO annotation. The genes upregulated by the expression of TREM2^WT^/TYROBP were significantly enriched (multiple testing corrected FET *p* value < 0.05) for pathways designated as “oxidoreductase activity, acting on paired donors, with incorporation or reduction of molecular oxygen,” “intracellular membrane-bounded organelle,” “electron carrier activity,” “heme binding,” “iron ion binding,” “oxidation-reduction process,” and “cellular response to heat” (Fig. 3b and Additional file 3: Table S2). In contrast, the genes downregulated by the expression of TREM2^WT^/TYROBP were enriched (corrected FET *p* < 0.05) in the pathways including “integral component of plasma membrane,” “extracellular region,” “extracellular matrix,” “structural constituent of chitin-based cuticle,” “extracellular space,” “potassium ion transport,” and “myosin light chain kinase activity” (Fig. 3b and Additional file 3: Table S2).

**Fig. 4:**
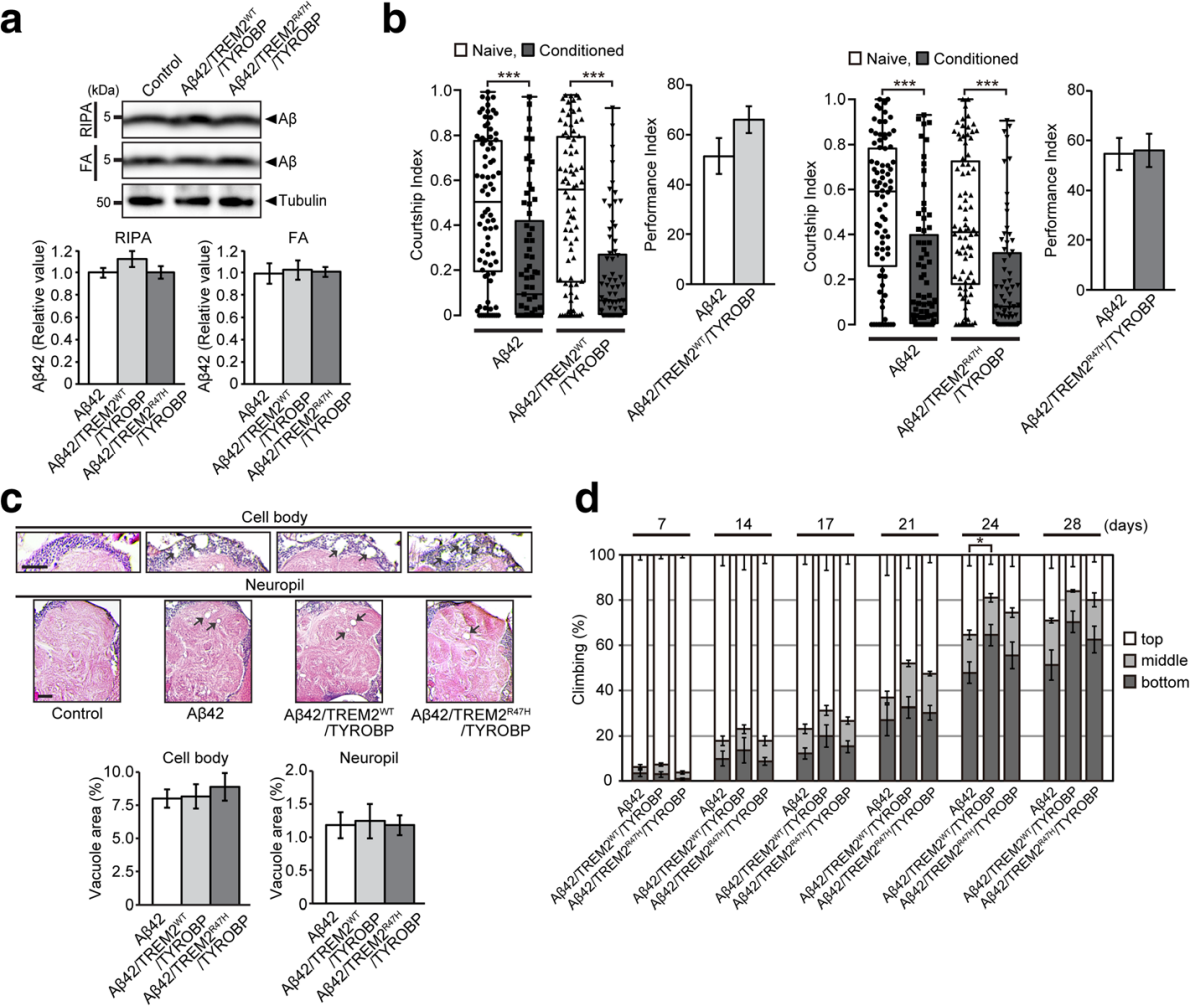
Effects of glial overexpression of TREM2/TYROBP on Aβ42 levels, Aβ42-mediated neurodegeneration, and Aβ42-induced behavioral deficits. **a** Western blotting of detergent-soluble (RIPA) and -insoluble/formic acid (FA) fractions from fly head lysates with neuronal expression of Aβ42 alone (Aβ42), neuronal expression of Aβ42 and glial expression of TREM2^WT^/TYROBP (Aβ42/TREM2^WT^/TYROBP), and neuronal expression of Aβ42 and glial expression of TREM2^R47H^/TYROBP (Aβ42/TREM2^R47H^/TYROBP) with anti-Aβ antibody. Tubulin was used as a loading control. **b** Courtship-conditioning assay. Courtship index values are represented by *box plot*. Performance indexes were calculated from courtship indexes. *n* = 67–80, ***p < 0.001, naïve vs conditioned by Mann–Whitney *U* test. Genotypes of flies are described in Additional file 2: Table S1. **c** Brain sections of flies with neuronal expression of Aβ42 alone (Aβ42), neuronal expression of Aβ42 and glial expression of TREM2^WT^/TYROBP (Aβ42/TREM2^WT^/TYROBP), and neuronal expression of Aβ42 and glial expression of TREM2^R47H^/TYROBP (Aβ42/TREM2^R47H^/TYROBP). Cell body regions (*top*) and neuropil regions (*middle*) of flies are shown. Control; Repo-LexA driver alone. Percentages of vacuole areas (indicated by *arrows*) in fly cortices are shown at the *bottom*. Scale bar: 100 μm. Mean ± SEM, *n* = 9–12 hemispheres. **d** Climbing assay. Average percentages of flies that climbed to the top (*white*) or middle (*light gray*), or stayed at the bottom (*dark gray*), of the vials. Ages (days after eclosion) are indicated on the *top* of the graph. Percentages of flies that stayed at the bottom were subjected to statistical analyses. Mean ± SEM, *n* = 5, *p < 0.05 by Student’s *t*-test

We next compared molecular pathways affected by glial expression of TREM2^WT^/TYROBP and those affected by neuronal expression of Aβ42 in fly brains. In mammals, the majority of Aβ peptides are produced from amyloid precursor protein (APP) in the late secretory pathway [62]. In our Aβ42 fly model, a signal sequence was fused to the N-terminus of Aβ42 [43] to target the peptide to the secretory pathway of neurons. Western blot analysis detected monomeric forms of Aβ42 as 4 kDa signals (Fig. 4a and [43]) and immunoprecipitation followed by mass spectrometry analysis confirmed that the fused signal peptide was correctly cleaved and intact Aβ42 peptides were produced [43]. Although this Aβ42 fly model directly expressed Aβ42 peptides in the endoplasmic reticulum, immuno-electron microscopy (Immuno-EM) detected Aβ42 signals in the secretory pathway, including ER, Golgi, and lysosomes [44], with minimal signals in the mitochondria and cytoplasm of neurons in Aβ42 fly brains. Moreover, secretion of Aβ peptides occurred in *Drosophila* cultured cells [44] and, in *Drosophila* brains, immuno-EM analysis occasionally detected Aβ42 accumulation in glial cells, suggesting that Aβ42 peptides were secreted from neurons and then taken up by glial cells [44]. The expression of Aβ42 in this model caused learning deficits followed by locomotor dysfunction and neurodegeneration with accumulation of detergent-insoluble Aβ42, in the brains. These results suggest that our Aβ42 fly model may recapitulate some aspects of Aβ42-mediated toxicity. Similar approaches have been utilized to generate transgenic Aβ42 fly models by other groups with consistent neurodegenerative phenotypes [63–65].

RNA sequence analysis in our Aβ42 fly model identified that neuronal expression of Aβ42 upregulated 437 genes and downregulated 485 genes in heads as compared to control flies (Fig. 3a, Additional file 2: Table S1). The upregulated DEGs were enriched in pathways including “endomembrane system,” “endoplasmic reticulum,” and “oxidation-reduction process” (Fig. 3b and Additional file 3: Table S2). By contrast, the downregulated DEGs were enriched in “electron carrier activity,” “oxidoreductase activity, acting on paired donors, with incorporation or reduction of molecular oxygen,” “intracellular membrane-bounded organelle,” “heme binding,” “oxidation-reduction process,” “iron ion binding,” “extracellular space,” “transferase activity, transferring phosphorus-containing groups,” “extracellular region,” “metabolic process,” “carboxylic ester hydrolase activity,” “cuticle pigmentation,” and “melanin biosynthetic process” (Fig. 3b and Additional file 3: Table S2).

Interestingly, this analysis revealed that eight of the 15 pathways (13 pathways for DEGs downregulated by Aβ42, three pathways for DEGs upregulated by Aβ42, one pathway is overlapped) enriched for the Aβ42 DEGs were also enriched for the TREM2^WT^/TYROBP DEGs in the same or opposite direction (Fig. 3b and Additional file 3: Table S2). For example, the pathways “extracellular space” and “extracellular region” were enriched for the genes downregulated by Aβ42 and by TREM2^WT^/TYROBP, while “oxidoreductase activity, acting on paired donors, with incorporation or reduction of molecular oxygen,” “oxidation-reduction process,” “electron carrier activity,” “heme binding,” “iron ion binding,” and “intracellular membrane-bounded organelle” were enriched for the genes downregulated by Aβ42 and for the genes upregulated by TREM2^WT^/TYROBP.

These unbiased analyses revealed that gene expression changes induced by neuronal expression of Aβ42 and glial expression of TREM2^WT^/TYROBP merged onto the same molecular pathways. However, in fly genome, there is no clear ortholog of either TREM2 or TYROBP. One possibility could be that glial cells sense Aβ42 and/or associated neuronal damages and then induce gene expression changes through endogenous signaling pathways. Ectopically expressed human TREM2/TYROBP may sense these damage-associated signals and impact the overlapping molecular pathways.

### TREM2^R47H^/TYROBP induces gene expression changes similar to those by TREM2^WT^/TYROBP in the fly brains

TREM2 variants were originally identified as causative mutations in patients with Nasu-Hakola disease [66]. However, recent genetic analysis revealed that R47H variant of TREM2 is associated with a three- to fourfold increased risk for AD [14, 15]. To examine the impact of glial expression of TREM2^R47H^/TYROBP in the fly brains at the molecular level, we generated RNA-seq data from the head samples in flies with the co-expression of TREM2^R47H^ and TYROBP (TREM2^R47H^/TYROBP) as described above [61]. Expression of TREM2^R47H^/TYROBP resulted in 290 upregulated genes and 365 downregulated ones (Fig. 3a and Additional file 2: Table S1). No significant alteration in either the size, gross morphology of brain structures, the numbers of glial cells, or neurons was observed in TREM2^R47H^ /TYROBP bigenic flies (Additional file 1: Figure S1), suggesting that gene expression changes induced by ectopic expression of TREM2^R47H^/TYROBP is not due to either structural changes or altered number of neurons or glial cells in the fly brain.

At the pathway level, FEA using GO annotation revealed that genes upregulated or downregulated by TREM2^R47H^/TYROBP were enriched in the same categories as those with TREM2^WT^/TYROBP (Fig. 3b and Additional file 3: Table S2), although some of the pathways that were enriched in the DEGs in TREM2^WT^/TYROBP, such as “iron ion binding,” “myosin light chain kinase activity,” and “cellular response to heat,” were not significantly enriched in the DEGs in TREM2^R47H^/TYROBP (Fig. 3b and Additional file 3: Table S2). The DEG signatures from TREM2^WT^/TYROBP and TREM2^R47H^/TYROBP flies shared about half of their members and that 98% of those overlapped genes changed in the same direction (corrected FET *p* = 2.0 × 10^−246^, 25.1-fold for downregulated genes; corrected FET *p* = 1.9 × 10^−133^, 27.4-fold for upregulated genes) (Fig. 3c).

To quantify differences in gene expression induced by TREM2^WT^/TYROBP and TREM2^R47H^/TYROBP, we directly compared mRNA expression levels between these two groups and identified 145 upregulated genes and 157 downregulated genes in TREM2^R47H^/TYROBP compared to TREM2^WT^/TYROBP (Fig. 3a). Interestingly, at an FDR of 5%, the upregulated DEGs are significantly enriched for “odorant binding,” “sensory perception of chemical stimulus,” “defense response,” and “response to pheromone,” suggesting that these functional pathways were activated by R47H mutation in TREM2 (Fig. 3b and Additional file 3: Table S2). Among these genes, *Drosophila* Toll-4 gene (the closest ortholog of human TLR7) detected in the “defense response” module is of particular interest, since TREM2 family proteins are known to modulate Toll-like receptor signaling in mammals [67, 68].

We also compared molecular pathways affected by neuronal expression of Aβ42 and those affected by glial expression of TREM2^R47H^/TYROBP (Fig. 3a, Additional file 2: Table S1). At the pathway levels, four of the above 15 pathways enriched in the Aβ42 DEGs were enriched in the TREM2^R47H^/TYROBP DEGs. At the gene level, a significant overlap between Aβ42 DEGs and TREM2 ^R47H^/TYROBP DEGs was observed (Fig. 3c, corrected *p* value ≤ 1.0 × 10^−32^, ≥ 7-fold).

Taken all together, biological pathways affected by glial expression of TREM2^WT^/TYROBP or TREM2^R47H^/TYROBP in fly heads are similar but about 300 genes show significant difference in mRNA expression. Moreover, glial expression of TREM2^R47H^/TYROBP impacts several common molecular pathways affected by neuronal expression of Aβ42, though TREM2^WT^/TYROBP appears to impact many more other common pathways affected by neuronal expression of Aβ42.

### Expression of TREM2/TYROBP in glial cells modifies molecular signatures induced by Aβ42 expression in neurons in fly brains

To investigate the effects of TREM2^WT^/TYROBP on phenotypes as well as gene expression signatures induced by Aβ42, we achieved neuronal expression of Aβ42 and glial expression of TREM2^WT^/TYROBP in fly brains by using two tissue-specific transgenes expression systems in *Drosophila* (Additional file 1: Figure S2A). Phenotypic characterization revealed that glial overexpression of TREM2^WT^/TYROBP did not affect either Aβ42 accumulation levels (Fig. 4a), courtship learning and memory (Fig. 4b), or Aβ42-mediated neurodegeneration (Fig. 4c); however, some exacerbation of Aβ42-mediated behavioral deficits was observed (Fig. 4d and Additional file 1: Figure S2B).

We next analyzed the effects of glial expression of TREM2^WT^/TYROBP on gene expression signatures induced by neuronal expression of Aβ42 in fly brains. RNA sequence analyses revealed that expression of Aβ42/TREM2^WT^/TYROBP resulted in upregulation of 533 genes and downregulation of 727 genes compared to control flies (Additional file 1: Figure S2C). Interestingly, comparison of FEA results between Aβ42/TREM2^WT^/TYROBP and Aβ42 flies revealed that seven of the 15 pathways enriched for the Aβ42 DEGs disappeared when TREM2^WT^/TYROBP was expressed in glia (Additional file 1: Figure S2D and Additional file 4: Table S3). These pathways include “electron carrier activity,” “oxidoreductase activity, acting on paired donors, with incorporation or reduction of molecular oxygen,” and “heme binding,” which were enriched in the DEGs downregulated by Aβ42 and in the DEGs upregulated by TREM2^WT^/TYROBP (Fig. 3b and Additional file 3: Table S2).

Using the same strategy, we also analyzed the effects of glial expression of TREM2^R47H^/TYROBP on phenotypes as well as gene expression signatures induced by neuronal expression of Aβ42 in fly brains (Additional file 1: Figure S2A). Similar to TREM2^WT^/TYROBP, glial overexpression of TREM2^R47H^/TYROBP did not affect Aβ42 accumulation levels (Fig. 4a), courtship learning and memory (Fig. 4b), or Aβ42-mediated neurodegeneration (Fig. 4c); although there is a trend toward subtle exacerbation of Aβ42-mediated behavioral deficits (Fig. 4d and Additional file 1: Figure S2B).

RNA sequence analyses revealed that expression of Aβ42/TREM2^R47H^/TYROBP resulted in upregulation of 661 genes and downregulation of 846 genes (Additional file 1: Figure S2C and Additional file 2: Table S1). Comparison of FEA results between Aβ42/TREM2^R47H^/TYROBP and Aβ42 flies revealed that nine of the 15 pathways enriched in the Aβ42 DEGs disappeared following glial expression of TREM2^R47H^/TYROBP (Additional file 1: Figure S2D and Additional file 4: Table S3). Among these nine pathways, six pathways were also disappeared following glial expression of TREM2^WT^/TYROBP, suggesting that the effects of TREM2^R47H^/TYROBP on Aβ42 were similar to that of TREM2^WT^/TYROBP by this analysis.

Taken together, glial expression of TREM2/TYROBP modifies molecular signatures induced by neuronal expression of Aβ42. Since glial expression of TREM2/TYROBP did not reduce Aβ42 levels (Fig. 4a), the observed changes in FEA results are not simply due to reduced response to Aβ42 in fly brains. In addition, since TREM2/TYROBP proteins are expressed in glial cells and Aβ42 peptides are expressed in neurons (Additional file 1: Figure S2A), this modulatory action likely reflects non-cell autonomous effects by TREM2/TYROBP.

### Neuronal Aβ42 and glial TREM2^R47H^/TYROBP synergistically downregulated genes associated with synaptic and immune function modules of the co-expressed gene networks from human AD brains

**Fig. 5:**
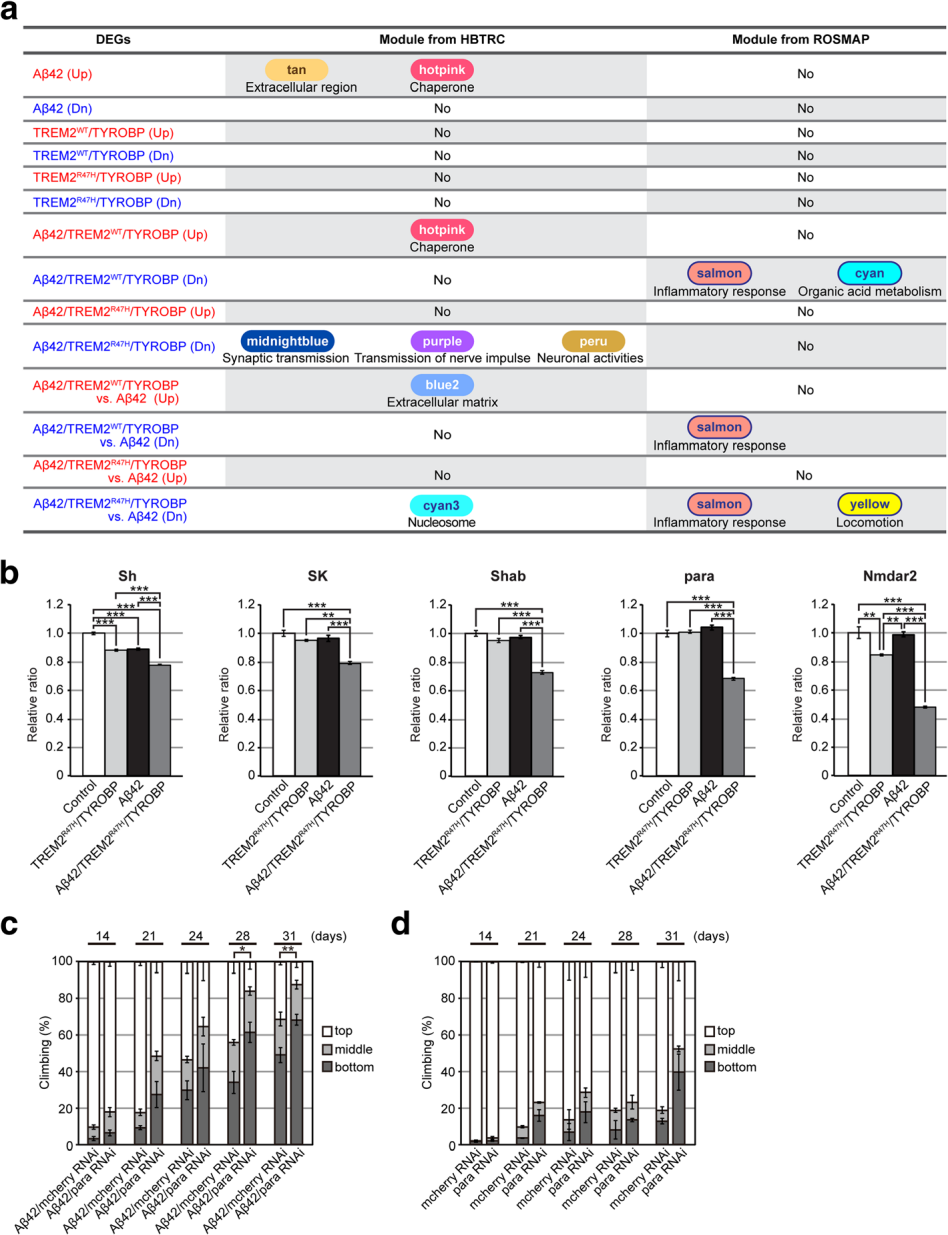
Gene regulatory network analysis of gene expression signatures in Aβ42/TREM2/TYROBP flies with human AD WGCNA. **a** Overlap between HBTRC or ROSMAP human AD WGCNA co-expression network modules and DEGs identified in (Fig. 3a and Additional file 1: Figure S2C). **b** mRNA expression levels of genes from “synaptic transmission” module validated by qPCR. mRNA levels in the heads of flies with neuronal expression of Aβ42 alone (Aβ42), glial expression of TREM2^R47H^/TYROBP (TREM2^R47H^/TYROBP) alone and neuronal expression of Aβ42 and glial expression of TREM2^R47H^/TYROBP (Aβ42/TREM2^R47H^/TYROBP) were analyzed by qRT-PCR. Control; drivers alone. Mean ± SEM, *n* = 4, *p < 0.05, **p < 0.01, and ***p < 0.001 by one-way ANOVA with post-hoc Tukey’s test. **c** Neuronal knockdown of para worsened Aβ42-induced locomotor deficits as revealed by climbing assay. **d** Neuronal knockdown of para by itself caused modest decline in locomotor functions upon aging. Mean ± SEM, *n* = 3–5, *p < 0.05 and **p < 0.01 by Student’s *t*-test. Genotypes of flies are described in Additional file 2: Table S1

Gene co-expression network analysis has uncovered a number of co-expressed gene modules pathologically related to human complex diseases including AD [11]. To investigate the relevance of the gene expression signatures in Aβ42, TREM2^WT^/TYROBP, TREM2^R47H^/TYROBP, Aβ42/TREM2^WT^/TYROBP, and Aβ42/TREM2^R47H^/TYROBP fly brains to AD, we investigated their association with the 111 co-expressed gene modules derived from co-regulation analyses of brain gene expression in the Harvard Brain Tissue Resource Center (HBTRC) AD and controls. The modules were annotated by the GO or pathways that the modules were enriched for. To do this, the fly DEGs were first converted to human orthologs by using DIOPT (DIOPT score > 1) [69]. The enrichment analysis shows that the genes upregulated by Aβ42 were enriched in the modules associated with “extracellular region” and “chaperone” (Fig. 5a and Additional file 5: Table S4a) while no module was enriched for the downregulated DEGs (Additional file 5: Table S4a). Neither the TREM2^WT^/TYROBP nor the TREM2^R47H^/TYROBP DEG signature showed significant enrichment in any of the HBTRC modules. By contrast, the DEGs upregulated by Aβ42/TREM2^WT^/TYROBP were enriched in the “chaperone” module (FET *p* = 0.022, 3.2-fold; Fig. 5a, Additional file 5: Table S4a) while Aβ42/TREM2^R47H^/TYROBP expression was associated with downregulation of genes enriched in synaptic transmission, neuronal activities, and transmission of nerve impulses, with FET *p* = 0.001 (2.1-fold), 0.024 (1.9-fold), and 0.024 (1.6-fold), respectively (Fig. 5a, Additional file 5: Table S4a). These results indicate that neuronal expression of Aβ42 and glial expression of TREM2^R47H^/TYROBP synergistically downregulated genes known to be associated with AD pathology [11], thus supporting the prediction that TREM2/TYROBP play roles in AD pathogenesis.

To validate these findings, we performed qPCR analyses in five DEGs from the “synaptic transmission” module with known functions related to neuronal activity; Sh, SK, Shab, para, and Nmdar2 (fly orthologs for potassium voltage-gated channel subfamily A, potassium calcium-activated channel subfamily N, potassium voltage-gated channel subfamily B, sodium voltage-gated channel alpha subunits, and glutamate ionotropic receptor NMDA type subunits, respectively). We found that expression levels of Sh were slightly downregulated by Aβ42 expression alone, while those of Sh and Nmdar2 were slightly downregulated by expression of TREM2^R47H^/TYROBP alone (Fig. 5b). By contrast, expression levels of all five genes were significantly downregulated in Aβ42/TREM2^R47H^/TYROBP flies.

We further examined whether downregulation of para, a fly ortholog for sodium voltage-gated channel alpha subunits, modifies neuronal dysfunction in Aβ42 flies. Neuronal knockdown of para by RNAi significantly worsened Aβ42-induced locomotor deficits (Fig. 5c). Moreover, neuronal knockdown of para by itself caused modest decline in locomotor functions in flies (Fig. 5d).

Taken altogether, these network analysis results suggest that genes associated with synaptic transmission were synergistically downregulated by co-expression of Aβ42 and TREM2^R47H^/TYROBP, which may lead to neuronal dysfunction.

To further explore the association of the DEG signatures with early phases of AD, we intersect them with the co-expression network modules from an independent cohort in the ROSMAP study. Again, the modules were annotated by the GO/pathways that the modules were most enriched for. The result is summarized in Fig. 5a and Additional file 5: Table S4b. The genes downregulated by Aβ42/TREM2^WT^/TYROBP were enriched for an “inflammatory response” module (salmon; corrected FET *p* = 0.005, 2.6-fold) and an “organic acid metabolism” module (cyan; corrected FET *p* = 0.014, 2.5-fold). Note that the “inflammatory response” module salmon was ranked number 7 in relation to AD pathology after ranking ROSMAP modules using multiple sorting features, including module-trait correlations and enrichment for genes correlated with or differentially expressed regarding neuropathological and clinical traits Braak staging, global cognition, CERAD neuropathological category (and, by extension, NIA-Reagan score). Importantly, this inflammatory module is not enriched for the DEGs by either Aβ42 alone or TREM2^WT^/TYROBP alone, suggesting interactions between Aβ42 and TREM2^WT^/TYROBP at the level of gene expression.

When gene expression levels were compared between Aβ42/TREM2/TYROBP and Aβ42 alone, the downregulated genes in Aβ42/TREM2^WT^/TYROBP were enriched in the “inflammatory response” module (salmon; corrected FET *p* = 0.015, 3.5-fold), while downregulated genes in Aβ42/TREM2^R47H^/TYROBP were enriched in the same “inflammatory response” module (salmon; corrected FET *p* = 5 × 10^−4^, 3.4-fold) and a “locomotion” module (yellow; ranked number 9; corrected FET *p* = 0.034, 1.9-fold).

In summary, for both TREM2^WT^/TYROBP and TREM2^R47H^/TYROBP, interaction with Aβ42 affected the “inflammatory response” pathway in flies. This is an interesting observation since neuroinflammation is implicated as a significant contributor to AD pathogenesis and is also consistent with the proposed anti-inflammatory consequences of TREM2 signaling in human microglia.

To test if the enrichment of synaptic transmission and inflammatory response modules was biased by the conserveness of these two pathways between fly and human, we analyzed the enrichment of known GO categories (based on the MSigDB gene sets) in the human orthologs of fly genes. As shown in Additional file 6: Table S5, the mostly enriched gene sets are big pathways including cytoplasm, metabolic process, nucleus, organelle part, and macromolecular complex, which account for 14.6%, 12.2%, 10%, 8.8%, and 7.2% of the 10,938 high confidence human orthologous genes (DIOPT score > 1; http://www.flyrnai.org/cgi-bin/DRSC_orthologs.pl) accordingly, with FDR adjusted FET *p* value < 1.2E-58. In contrast, the immune system genes only account for < 1.1% of the orthologs and were not enriched (FET *p* value ≥ 0.54), while synaptic transmission accounted for 1.0% of the orthologs and was marginally enriched (FET *p* value = 0.004). Thus, it is unlikely that the significant correlation with the inflammatory and synaptic modules in fly signatures were caused by an artifact of overrepresentation of these pathways in the fly-human orthologous genes.

In summary, since Aβ42 accumulation, TREM2/TYROBP activation, altered inflammatory response, and synaptic dysfunctions are all implicated in early phases of AD pathogenesis, Aβ42/TREM2/TYROBP flies may recapitulate some molecular signatures relevant to early stages of AD.

### Molecular pathways affected by neuronal expression of tau do not overlap with those affected by glial expression of TREM2/TYROBP in fly brains

In the pathogenesis of AD, abnormal accumulation and toxicity of tau is believed to play a critical role in neurodegeneration. Thus, identification of molecular signatures induced by simultaneous activation of TREM2/TYROBP axis and accumulation of tau may provide important information underlying neurodegenerative process in AD.

We first compared molecular pathways affected by neuronal expression of tau and those affected by glial expression of TREM2/TYROBP in fly heads. We performed RNA sequence analyses and characterized gene expression signatures using an established fly model of human tau toxicity [70] in which expression of human tau causes progressive degeneration of photoreceptor neurons in the retina [71]. Expression of tau in photoreceptor neurons using GMR-GAL4 driver upregulated 384 genes and downregulated 418 genes in the heads compared to control flies (Additional file 1: Figure S3A and Additional file 2: Table S1). The upregulated DEGs in tau fly heads were associated with “endosome transport via multivesicular body sorting pathway,” “ESCRT III complex,” and “vacuolar transport” (Additional file 1: Figure S3B and Additional file 7: Table S6). In contrast, downregulated DEGs in tau fly heads were significantly enriched in “rhabdomere” and “striated muscle thin filament” (Additional file 1: Figure S3B and Additional file 7: Table S6).

We also analyzed gene expression changes caused by pan-glial expression of TREM2^WT^/TYROBP or TREM2^R47H^/TYROBP in the same genetic background carrying the GMR-GAL4 driver. Glial expression of TREM2^WT^/TYROBP upregulated 448 genes and downregulated 306 genes while TREM2^R47H^/TYROBP upregulated 475 genes and downregulated 426 genes (Additional file 1: Figure S3A and Additional file 2: Table S1). There were 29–52 genes common between tau DEGs and TREM2/TYROBP DEGs (Additional file 1: Figure S3C, corrected *p* value ≤ 10^−10^, ≥ 3.9-fold). However, we observed no pathway that was enriched in both DEG signatures (Additional file 1: Figure S3B and Additional file 7: Table S6).

Taken together, these results suggest that molecular signatures induced by expression of tau are dissimilar to those induced by TREM2/TYROBP in fly heads at the functional pathway level.

### Glial expression of TREM2/TYROBP exacerbated tau-mediated neurodegeneration

Next, we examined the effects of glial expression of TREM2/TYROBP on gene expression signatures as well as neurodegenerative phenotypes induced by tau expression. In order to achieve expression of tau in photoreceptor neurons and expression of the TREM2/TYROBP complex in glial cells simultaneously, we utilized two tissue-specific transgenes expression systems in *Drosophila* (Additional file 1: Figure S4A).

**Fig. 6:**
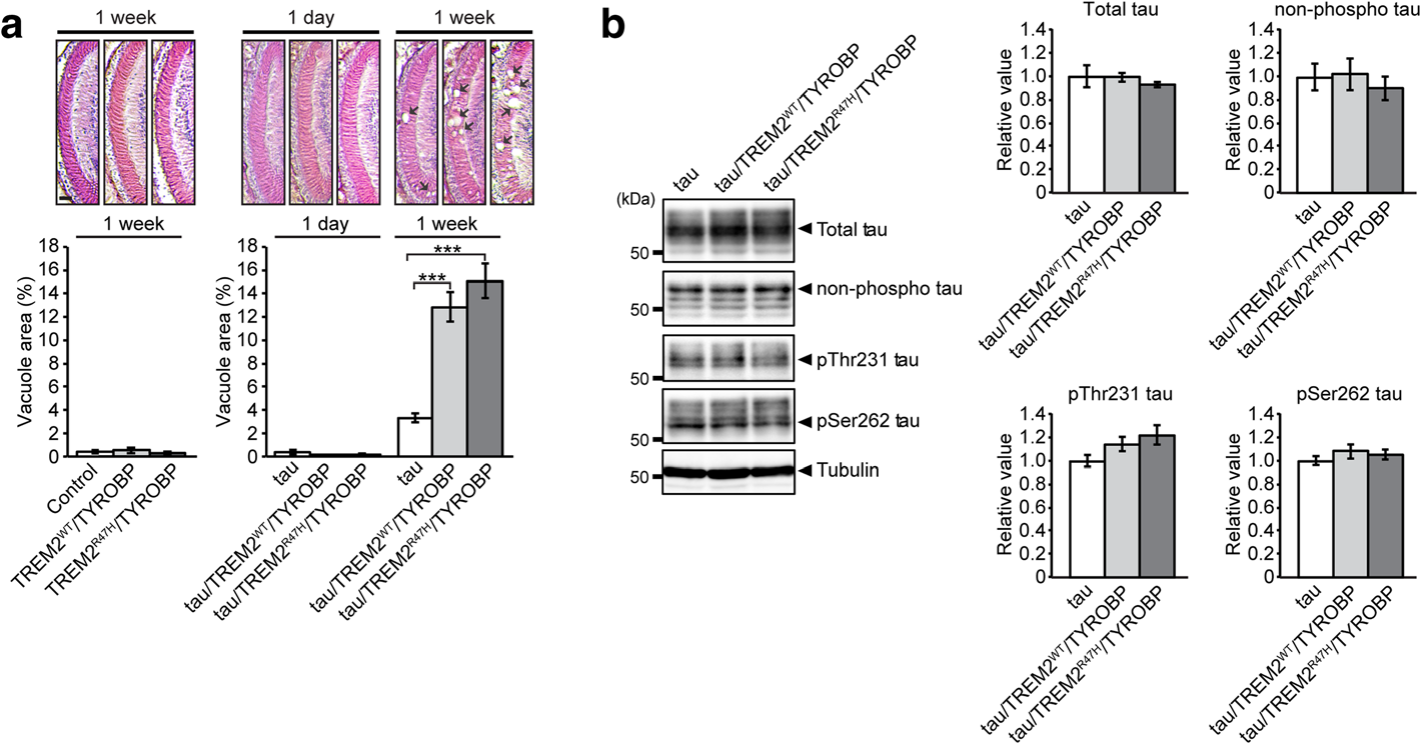
Glial expression of TREM2/TYROBP exacerbated tau-mediated neurodegeneration without altering tau phosphorylation levels. **a** *Top:* The lamina of control flies carrying drivers alone (control), flies with glial expression of TREM2^WT^/TYROBP alone (TREM2^WT^/TYROBP), flies with glial expression of TREM2^R47H^/TYROBP alone (TREM2^R47H^/TYROBP), flies expressing tau alone (tau), expression of tau and glial expression of TREM2^WT^/TYROBP (Tau/TREM2^WT^/TYROBP), and expression of tau and glial expression of TREM2^R47H^/TYROBP (tau/TREM2^R47H^/TYROBP). Flies are 1 day or 1 week after eclosion. *Bottom:* Quantification of neurodegeneration. Mean ± SEM, *n* = 12–18 hemispheres. ***p < 0.001, Student’s *t*-test. **b** Western blot analysis of fly head lysates with antibody against total tau and an antibody that recognizes tau without phosphorylation at Ser194, 195, 198, and 202 (non-phospho Tau), tau phosphorylated at Thr231 (pThr231 Tau), and tau phosphorylated at Ser262 (pSer262 Tau). Tubulin was used as a loading control. Representative blots are shown in the *left* and quantification in the *right*. Mean ± SEM, *n* = 4. Genotypes of flies are described in Additional file 2: Table S1

Expression of human tau in photoreceptor neurons causes progressive neurodegeneration in the lamina [71], the first synaptic neuropil of the optic lobe containing photoreceptor axons and abundant glial cells [72]. We observed that pan-glial expression of both TREM2^WT^/TYROBP and TREM2^R47H^/TYROBP significantly exacerbated this neurodegeneration, while pan-glial expression of TREM2/TYROBP alone (i.e. in the absence of neuronal tau expression) did not show neurodegeneration (Fig. 6a). We also examined whether glial expression of TREM2/TYROBP increased the levels of tau and/or tau phosphorylated at AD-related sites. Western blot analyses with pan-tau or phospho-tau specific antibodies did not detect significant increase in either tau levels or phosphorylation status of tau by glial expression of TREM2/TYROBP (Fig. 6b). These results suggest that glial expression of TREM2/TYROBP exacerbates tau-mediated neurodegeneration without affecting tau accumulation or phosphorylation status, consistent with recent report using TREM2 deficiency mice [73].

### Analysis of the gene regulatory network in AD brains revealed that tau and TREM2/TYROBP synergistically downregulated genes overrepresented in the modules related to immune systems associated with AD pathogenesis

We generated RNA-seq data from tau/TREM2^WT^/TYROBP and tau/TREM2^R47H^/TYROBP flies and identified gene expression signatures in comparison with control flies. Expression of tau/TREM2^WT^/TYROBP upregulated 377 genes and downregulated 476 genes, while expression of tau/TREM2^R47H^/TYROBP upregulated 596 genes and downregulated 601 genes (Additional file 1: Figure S4B and Additional file 2: Table S1).

Most of the pathways enriched in these DEGs (Additional file 1: Figure S4C and Additional file 8: Table S7) were the same as those detected in either TREM2/TYROBP alone or tau alone (Additional file 1: Figure S3B and Additional file 7: Table S6). However, we observed that “proteolysis” and “UDP-glycosyltransferase activity” were uniquely enriched in the DEGs downregulated by tau/TREM2^WT^/TYROBP and tau/TREM2^R47H^/TYROBP, respectively (Additional file 1: Figure S3B and Additional file 8: Table S7). The “proteolysis” pathway contains proteases including angiotensin-converting enzyme (ACE), which have been associated with AD [74], and UDP-glycosyltransferases, enzymes associated with oligodendrocyte myelination, disruption of which has been implicated in neurodegeneration in AD [12].

To further explore the relevance of the gene expression signatures in tau, TREM2^WT^/TYROBP, TREM2^R47H^/TYROBP, tau/TREM2^WT^/TYROBP, and tau/TREM2^R47H^/TYROBP flies to AD, we investigated their association with the 111 co-expressed gene modules derived from co-regulation analyses of brain gene expression in the HBTRC AD and controls [11]. The enrichment analysis shows that no module was enriched for tau DEG signature. Moreover, neither the TREM2^WT^/TYROBP nor the TREM2^R47H^/TYROBP DEG signature showed significant enrichment in any of the HBTRC modules.

**Fig. 7:**
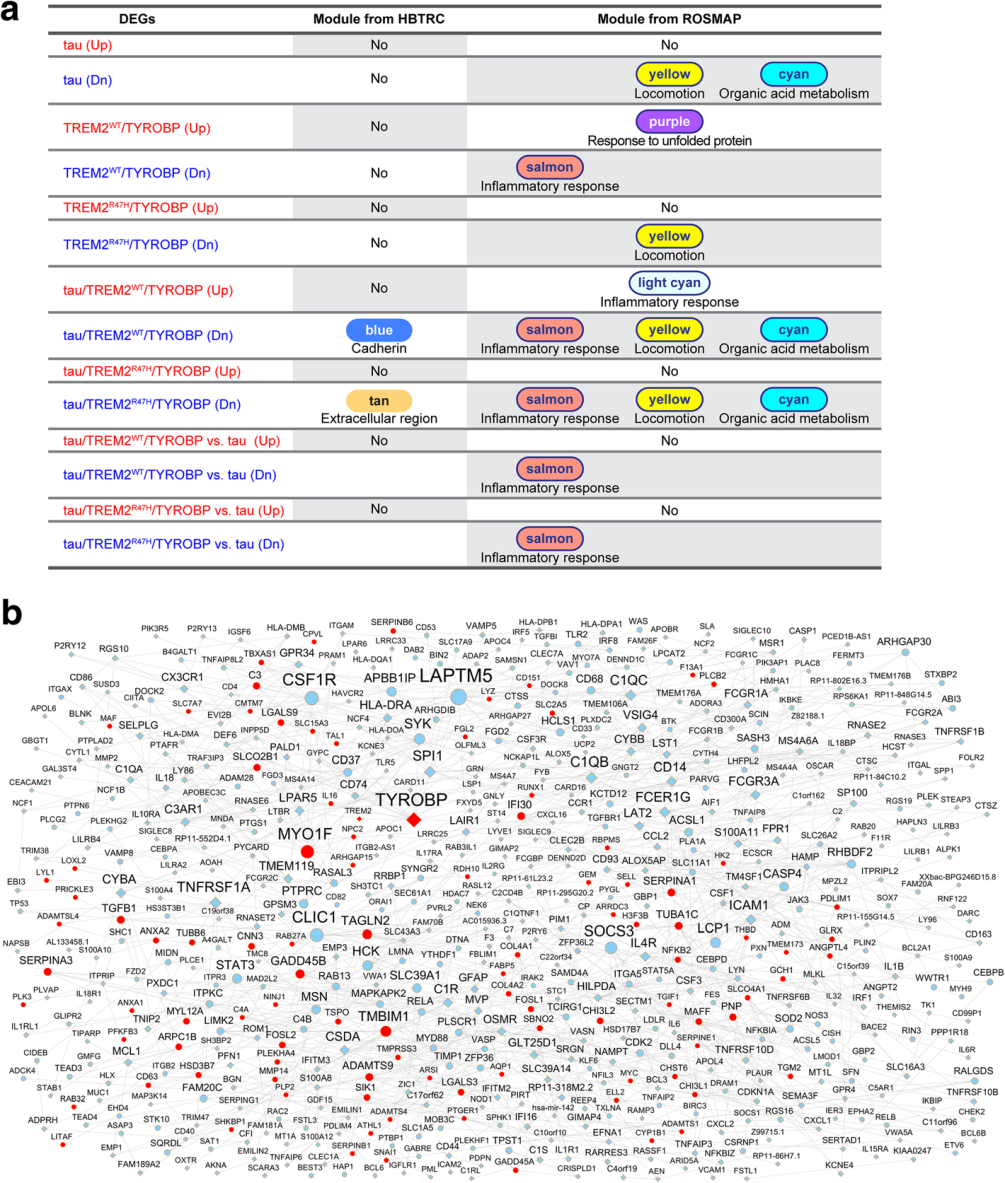
Gene regulatory network analysis of gene expression signatures in tau/TREM2/TYROBP flies with human AD WGCNA. **a** Overlap between HBTRC or ROSMAP human AD WGCNA co-expression network modules and DEGs identified in (Additional file 1: Figures S3A and S4B). **b** Causal regulatory network of the genes from two inflammatory response modules “salmon” and “lightcyan” identified from the ROSMAP human AD WGCNA co-expression network. DEGs in at least one of the present fly transgenic models are denoted by *red color*, otherwise by *cyan color*. Genes having fly orthologs are in *eclipse shape*, otherwise in *diamond shape*. Node size is proportional to the number of downstream genes. Genotypes of flies are described in Additional file 2: Table S1

By contrast, the downregulated DEG signatures in tau/TREM2^WT^/TYROBP and tau/TREM2^R47H^/TYROBP were enriched in the “cadherin” module (corrected FET *p* = 0.035, 1.6-fold; Fig. 7a, Additional file 9: Table S8a) and the “extracellular region” module (corrected FET *p* = 0.023, 2.0-fold; Fig. 7a, Additional file 9: Table S8a), respectively. Since these two modules are predicted to be highly associated with AD pathology [11], our data suggest pathological interactions between tau and TREM2/TYROBP at the level of gene expression in flies.

In the co-expression network from ROSMAP, the DEGs downregulated by tau/TREM2^WT^/TYROBP and tau/TREM2^R47H^/TYROBP significantly overlapped with three modules (Fig. 7a, Additional file 9: Table S8b): the “inflammatory response” module (salmon; ranked number 7) (corrected FET *p* = 9.0 × 10^−4^, 3.3-fold and corrected FET *p* = 3.7 × 10^−4^, 3.0-fold, respectively); “locomotion” (yellow; ranked number 9) (corrected FET *p* = 4.4 × 10^−3^, 2.1-fold and corrected FET *p* = 0.04, 1.7-fold, respectively); and “organic acid metabolism” (cyan; ranked number 24) (corrected FET *p* = 5.7 × 10^−3^, 3.1-fold and corrected FET *p* = 0.02, 2.5-fold, respectively). These three modules also significantly overlapped with the Aβ42-related DEG signatures, as described above (Fig. 5a). The “locomotion” and “organic acid metabolism” modules were also enriched for DEGs downregulated by expression of tau alone (corrected FET *p* = 0.01, 2.0-fold and corrected FET *p* = 0.02, 2.8-fold, respectively), while the “inflammatory response” module salmon was enriched in the DEGs downregulated by expression of TREM2^WT^/TYROBP alone (corrected FET *p* = 4.1 × 10^−5^, 4.9-fold). The “inflammatory response” module (or the salmon module, ranked number 7) was also enriched in the DEGs from downregulated by tau/TREM2^WT^/TYROBP in comparison with tau (corrected FET *p* = 0.01, 3.6-fold), or by tau/TREM2^R47H^/TYROBP in comparison with tau (corrected FET *p* = 0.006, 3.4-fold). In the ROSMAP data (Additional file 10: Table S9), this salmon module had three members downregulated in AD brains, including *GADD45A*, *FABP5*, and *BAALC-AS1*, the first two of which were also downregulated in the present tau/TREM2^WT^/TYROBP and tau/TREM2^R47H^/ TYROBP flies (FET *p* = 9.2 × 10^−5^, 14.7-fold), consistent with the existence of substantial network consistency when human data and fly data are compared.

Of particular interest, the DEGs upregulated by tau/TREM2^WT^/TYROBP were enriched for another “inflammatory response” module (lightcyan; ranked number 8; corrected FET *p* = 0.04, 2.9-fold) in the ROSMAP network. This lightcyan module contained five AD GWAS loci, including *CD33*, *INPP5D*, *MS4A4A*/*MS4A6A*, *RIN3*, and *TREM2* (Additional file 10: Table S9). Moreover, *TYROBP* was a member of this ROSMAP lightcyan module. This module was not enriched with DEGs upregulated by either tau alone or TREM2^WT^/TYROBP alone, suggesting that genetic interactions between tau and TREM2^WT^/TYROBP may induce this gene expression signature. Moreover, significant enrichment was not observed with the DEG signatures in tau/TREM2^R47H^/TYROBP flies, suggesting that the TREM2^R47H^ variant may have weaker impact on this module than does TREM2^WT^.

Taken together, these results revealed that different components of the immune response system were either activated or inhibited by the tau/TREM2/TYROBP pathway. Since both the salmon and lightcyan modules were highly ranked for their predicted relationship to AD pathology, the present results highlighted the importance of inflammatory response subnetworks as potential targets for disease intervention.

As shown in Additional file 1: Figure S5, the salmon and lightcyan modules in the ROSMAP network were adjacent to each other in the cluster dendrogram, indicating that the two inflammatory response modules were highly related in the human data, even though they were regulated differently in tau/TREM2/TYROBP flies. Therefore, our fly models provide valuable biological insights into the human data that were not otherwise evident. To investigate the causal regulatory relationships among the inflammatory response module genes, we combined the genes from the two inflammatory response modules and overlaid the combined gene set onto a Bayesian causal network constructed from the ROSMAP data by using an approach described in our previous study [11]. Figure 7b shows the network structure of the 604 inflammatory response genes of which 270 genes have fly orthologs. Over one-third (96) of the 270 fly orthologs were differentially expressed in at least one of the fly transgenic models analyzed here, resulting in a 1.3-fold enrichment (*p* value = 2.5 × 10^−4^). *TYROBP* was highlighted as a key regulator for controlling a large number of downstream genes in this inflammatory response network: 17, 36, and 79 genes were in the immediate first, second, and third layer downstream of TYROBP, respectively. Therefore, the causal network analysis further validated the causal role of TYROBP and informed other novel key regulators, which modulate the inflammatory response pathways, such as, *LAPTM5*, *MYO1F*, *CLIC1*, and *CSF1R*, which were highlighted by a large node size in Fig. 7b.

## Discussion

There is an increasing appreciation that immunological mechanisms play important roles in AD pathogenesis, as evidenced by the identification of a number of genes expressed in immune cells of the central nervous system (CNS) carrying genetic variants associated with increased risk for late-onset AD, including *CD33* [75], *TREM2* [14, 15], and *CR1* [76]. Thus, dysregulation of immune response genes and/or pathways are believed to be key factors in the cause and/or progression of AD. The present transcriptomic analysis indicated an overlap between glial expression of TREM2/TYROBP and neuronal expression of Aβ42. FEA of the DEGs suggested a strong overlap of the common pathways regulated by TREM2^WT^/TYROBP and by Aβ42. More than half of the pathways detected in Aβ42 DEGs were also detected in TREM2^WT^/TYROBP DEGs with the same or opposite regulation direction (Fig. 3b). In addition, more than half of the pathways regulated by Aβ42 disappeared by glial expression of TREM2^WT^/TYROBP (Additional file 1: Figure S2D), suggesting that the changes in the TREM2^WT^/TYROBP signaling pathway might represent a defense reaction to Aβ42 toxicity. This is consistent with the proposed role of TREM2 as a component of the microglial reaction to Aβ-related pathology [18–20].

In order to identify potential modules associated with AD pathogenesis, we overlaid DEGs onto two independent human AD co-expression networks, one from the HBTRC AD cohort and the other from the ROSMAP AD cohort. Synaptic transmission modules from the HBTRC network and inflammatory response modules from the ROSMAP network were among the most interesting subnetworks enriched with different sets of DEGs (Fig. 5a). Different modules emerged from the two networks, possibly due to differences in the distribution of AD severity within each cohort. The ROSMAP cohort contains normal individuals, and patients with mild cognitive impairment (MCI) to mild to moderate stages of dementia with very few individuals with advanced dementia [41, 42], while the HBTRC AD cohort samples were concentrated in more advanced stages of the disease (CDR 3.0 and higher [11]).

When TREM2/TYROBP is expressed in glial cells, three out of the five ROSMAP modules enriched with the DEGs identified from fly models of tau toxicity were also enriched with the DEGs detected from fly models of Aβ42 toxicity (Figs. 5a and 7a). This suggests that changes in these pathways maybe part of the pathological interaction between Aβ and tau toxicity and therefore may have implications for elucidation of the pathogenesis of early phases of AD. In turn, identification of molecules that play roles in early phases of AD may point to novel sites of intervention where progression of AD may be slowed or arrested.

We also found that, while the “inflammatory response” module salmon was enriched in downregulated DEGs from both Aβ42/TREM2^WT^/TYROBP and tau/TREM2^WT^/TYROBP genotypes, the “inflammatory response” lightcyan module was enriched in upregulated DEGs from the tau/TREM2^WT^/TYROBP genotype (Fig. 7a). Activation of this subnetwork of the inflammatory response pathway may represent an event linked to late stages of AD characterized by tau toxicity and upregulation of TREM2/TYROBP signaling. Interestingly, the lightcyan module was not detected in the tau/TREM2^R47H^/TYROBP genotype, suggesting that the pathogenic R47H variant may have a negative impact on activating this inflammatory response. The finding has significant implication for selectively and differentially targeting subnetworks of inflammatory response for potential therapeutic intervention. The lightcyan module contained *TYROBP*, as well as several AD GWAS gene loci, including *CD33, INPP5D, MS4A4A/MS4A6A, RIN3, and TREM2*. In addition, this module was highly enriched for various AD signatures and hence was the top ranked module in relation to AD pathology among all ROSMAP modules. Taken together, these results highlighted the lightcyan module as an interesting target for potential disease intervention from both genetic and the molecular pathway perspectives.

Innate immune response is a conserved biological process that multicellular organisms use for their defense against pathogens and toxic stimuli. In AD brains, there is a sustained increase in innate immune activity. In fruit fly, immune response relies on combined action of both cellular processes, such as the phagocytosis of invading microbials, and humoral immune responses, such as the secretion of antimicrobial peptides (AMPs) into the hemolymph [77]. NF-κB signaling pathways play paramount roles in modulating humoral immune response. We noted that DEGs in the present Aβ or Tau flies with or without TREM2/TYROBP showed a significant overlap with the fly Rel/NF-κB perturbation signatures induced by *Rel* mutation or *Rel* overexpression in Pal *et al*. [78] (Additional file 11: Table S10a). In addition, we found that *Rel* signatures were enriched for the immune response modules in both HBTRC and ROSMAP data (Additional file 11: Table S10b). For example, the *Rel* overexpression genes were enriched for the “yellow” (response to biotic stimulus) module in the HBTRC dataset (2.5-fold, BH adjusted FET *p* value 0.035) and the “salmon” (inflammatory response) module in the ROSMAP dataset (6.4-fold, BH adjusted FET *p* value 0.001). This suggests that fly is a promising model for studying the NF-κB-controlled immune signaling pathways that are implicated in the Aβ or tau pathologies of AD.

Besides the impact on the immune response modules, we systematically examined the impact of the *TREM2*^*R47H*^ variant on neuronal expression of Aβ42 or tau. Overall, the gene expression changes induced by *TREM2*^*R47H*^ were similar to those induced by *TREM2*^*WT*^ in terms of GO function and co-expression network enrichment under conditions with or without Aβ42 or tau. However, we noted a significant enrichment for the synaptic transmission modules with downregulated DEGs in Aβ42/TREM2^R47H^/TYROBP flies, but not with Aβ42/TREM2^WT^/TYROBP flies (Fig. 5a and Additional file 5: Table S4a), suggesting a potential role of R47H variant involved in dysregulation of neuronal activities. Direct comparison of mRNA levels between *TREM2*^*R47H*^ and *TREM2*^*WT*^ under various genotype configurations revealed several consistent GO categories, including upregulation of “odorant binding,” “extracellular region,” “defense response,” and “response to pheromone,” downregulation of “phosphatidate phosphatase activity.” Among these categories, “extracellular region” was consistently identified to differ except under the tau expression background. “Extracellular region” is of particular interest because the R47H variant is located in the extracellular region of TREM2 protein. It is postulated that the amino acid change by this mutation interferes the normal biological function of TREM2, such as the binding to its ligands, its receptor function and its processing by proteases, leading to impaired biological pathways implicated in the pathogenesis of AD [79]. In addition, “defense response” contains Toll-4, a fly ortholog of mammalian TLR7, suggesting that TREM2 R47H variant may have distinct impact on Toll-like receptor singling. We anticipate that the gene signatures and pathways identified in this study will be a starting point for a complete identification of the exact molecular mechanisms underlying how risk for AD is specified by *TREM2*^*R47H*^.

## Conclusions

In summary, we constructed novel transgenic fly models of AD in order to study the genetic interactions between glial expression of TREM2/TYROBP and the neuronal expression of Aβ42 or tau, the two hallmark proteins for the characterization of AD neuropathology. Using these novel transgenic fly models of AD, we also investigated the impact of a TREM2 pathogenic R47H variant (rs75932628), for which the observed effect size has been estimated to be comparable to that of the *APOE ε4* allele [15]. To the best of our knowledge, we are the first to systematically analyze phenotypic and genome-wide gene expression changes associated with overexpression of the WT and R47H mutant type TREM2/TYROBP and their interaction with Aβ42- or tau-related pathobiology *in vivo*. A recent work reports that R47H mutation impairs TREM2-mediated microglial response to Aβ pathology [34], while our results demonstrate that *TREM2*^*R47H*^ is capable of promoting tau-mediated neurodegeneration. The comprehensive pathological and molecular data generated through this study strongly validate the causal role of *TREM2/TYROBP* in driving molecular networks in AD and AD-related phenotypes in flies and also provides insight into the role of R47H variant TREM2 in AD pathogenesis.

## Additional files

Additional file 1: **Figure S1.** No significant alteration in either the gross morphology of brain structures or the number of neuronal and glial cells was observed in TREM2/TYROBP flies. **Figure S2.** Molecular pathways affected by neuronal expression of Aβ42 and glial expression of TREM2/TYROBP. **Figure S3.** Molecular pathways affected by tau do not overlap with those affected by glial TREM2/TYROBP. **Figure S4.** Gene expression signatures in tau/TREM2/TYROBP flies. **Figure S5.** Heatmap showing the topological overlapping matrix (TOM) from weighted gene co-expression network analysis. **Table S11.** Primer sequences for RT-PCR and qRT-PCR. (PDF 4937 kb)

Additional file 2: **Table S1.** Differentially expressed genes identified from different comparisons under FDR ≤ 0.05 and absolute log2 fold change ≥ 1.2. (XLSX 1236 kb)

Additional file 3: **Table S2.** Functional enrichment of DEGs identified in TREM2^WT^/TYROBP, TREM2^R47H^/TYROBP, and Aß42 files. (XLSX 23 kb)

Additional file 4: **Table S3.** Functional enrichment of DEGs identified in Aβ42, Aβ42/TREM2^WT^/TYROBP, and Aβ42/TREM2^R47H^/TYROBP files. (XLSX 23 kb)

Additional file 5: **Table S4a.** Overlap between HBTRC human AD co-expression network modules and DEGs identified in Aβ42, Aβ42/TREM2^WT^/TYROBP. **Table S4b.** Overlap between ROSMAP human AD co-expression network modules and DEGs identified in Aβ42, Aβ42/TREM2^WT^/TYROBP. (XLSX 18 kb)

Additional file 6: **Table S5.** Overlap between MSigDB gene ontology/pathway gene sets and fly-human conserved genes. (XLSX 299 kb)

Additional file 7: **Table S6.** Functional enrichment of DEGs identified in TREM2^WT^/TYROBP, TREM2^R47H^/TYROBP, and Tau files. (XLSX 18 kb)

Additional file 8: **Table S7.** Functional enrichment of DEGs identified in Tau/TREM2^WT^/TYROBP, Tau/TREM2^R47H^/TYROBP files. (XLSX 23 kb)

Additional file 9: **Table S8a.** Overlap between HBTRC human AD co-expression network modules and DEGs identified in Tau/TREM2^WT^/TYROBP and Tau/TREM2^R47H^/TYROBP files. **Table S8b.** Overlap between ROSMAP human AD co-expression network modules and DEGs identified in Tau, Tau/TREM2^WT^/TYROBP, and Tau/TREM2^R47H^/TYROBP files. (XLSX 18 kb)

Additional file 10: **Table S9** Module membership from weighted gene co-expression network analysis for ROSMAP gene expression data. (XLSX 456 kb)

Additional file 11: **Table S10a.** Overlap between fly Rel mutation or overexpression signatures and DEGs in Aβ and Tau flies. **Table S10b.** Overlap between fly Rel mutation or Rel overexpression signatures and human AD network modules. (XLSX 13 kb)
