# Supplementary material for: Integrated biology approach reveals molecular and pathological interactions among Alzheimer’s Aβ42, Tau, TREM2, and TYROBP in Drosophila models
Source: Genome Med. 2018 Mar 29;10:26. doi: 10.1186/s13073-018-0530-9 (PMC5875009; doi:10.1186/s13073-018-0530-9)
Supplement: Supplementary file 1 — Figure S1. No significant alteration in either the gross morphology of brain structures or the number of neuronal and glial cells was observed in TREM2/TYROBP flies. Figure S2. Molecular pathways affected by neuronal expression of Aβ42 and glial expression of TREM2/TYROBP. Figure S3. Molecular pathways affected by tau do not overlap with those affected by glial TREM2/TYROBP. Figure S4. Gene expression signatures in tau/TREM2/TYROBP flies. Figure S5. Heatmap showing the topological overlapping matrix (TOM) from weighted gene co-expression network analysis. Table S11. Primer sequences for RT-PCR and qRT-PCR. (PDF 4937 kb) [file 13073_2018_530_MOESM1_ESM.pdf]

Additional file 1: Figure S1

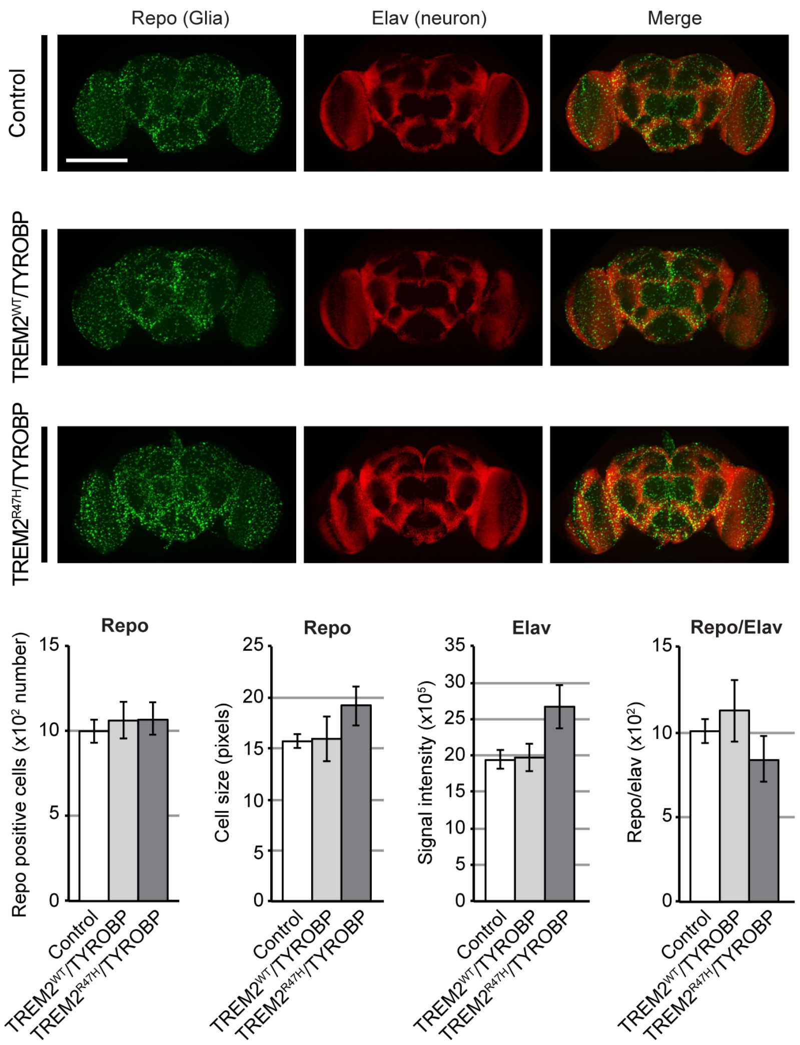

Figure S1. No significant alteration in either the gross morphology of brain structures or the number of neuronal and glial cells was observed in TREM2/TYROBP flies.

(Top) Fly brains co-expressing $TREM2^{WT}$ or $TREM2^{R47H}$ with TYROBP driven by Repo-LexA were double stained with anti-Repo and anti-elav antibody. Representative images were captured from anterior side and stacked. Repo-positive glial cells (green) and elav-positive neurons (red) in the fly brain at 7-day-old were shown. Scale bar: 200 μm. (Bottom) The number and size of Repo-positive glial cells and signal intensity of elav-positive neurons were quantified. Control; Repo-LexA driver alone. n = 6 brains (6 sections/brain), no significant difference by Student's *t*-test.

Additional file 1: Figure S2

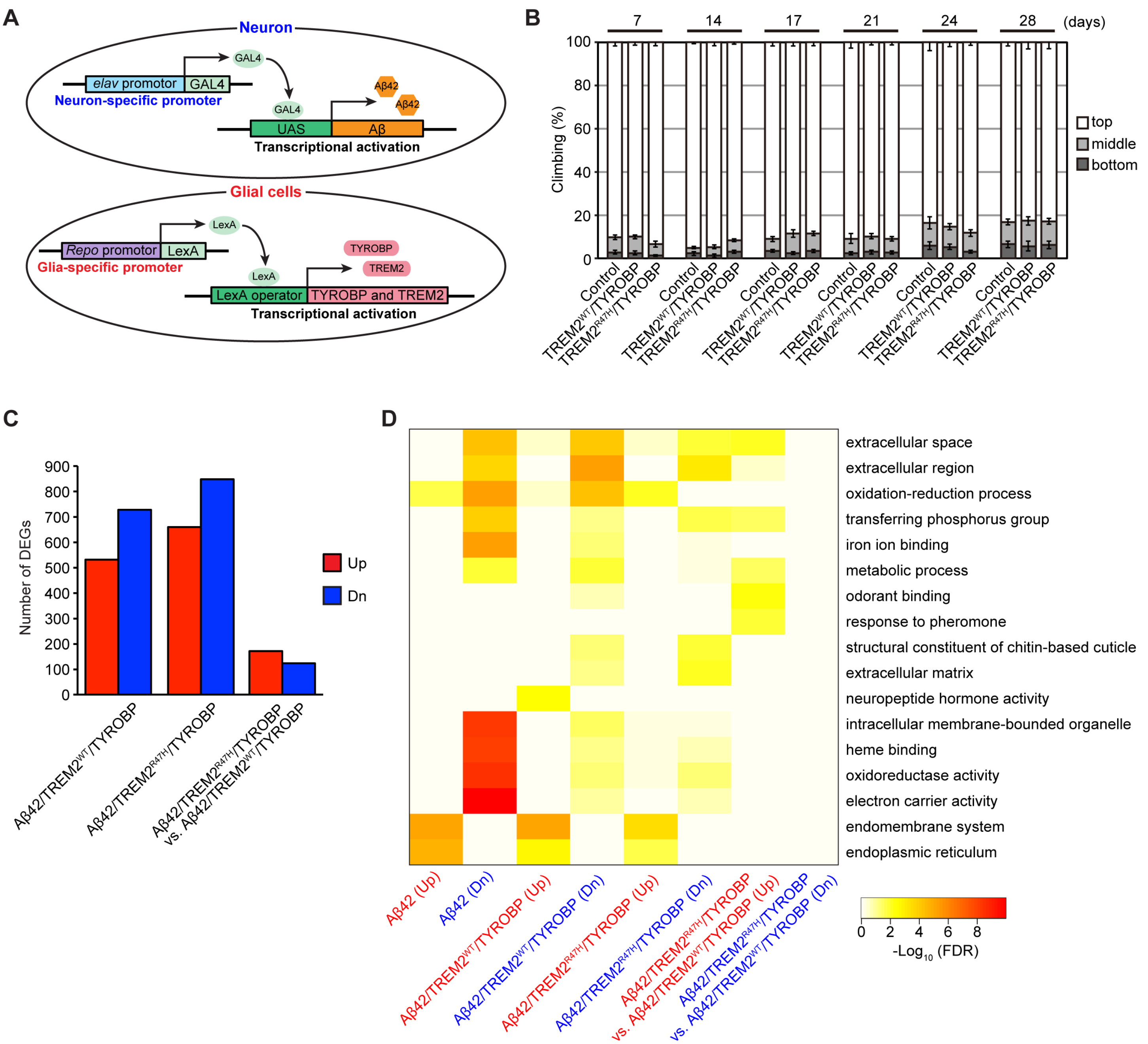

Figure S2. Molecular pathways affected by neuronal expression of Aβ42 and glial expression of TREM2/TYROBP.

(**A**) Schematic representation of the transgene expression system of Aβ42 and TREM2/TYROBP in *Drosophila*. Human Aβ42 was expressed in neurons by GAL4-UAS system with pan-neuronal elav-GAL4 driver, while human TREM2/TYROBP complex was expressed in glial cells by LexA system with pan-glial Repo-LexA driver. (**B**) Glial expression of TREM2/TYROBP did not affect locomotor activity of flies during aging measured by

Additional file 1: Figure S2

climbing assay. Average percentages of flies that climbed to the top (white) or middle (light gray), or stayed at the bottom (dark gray) of the vials. Ages (days after eclosion) are indicated on the top of the graph. Percentages of flies that stayed at the bottom were subjected to statistical analyses. Mean ± SEM, n = 5, no significant difference by Student's *t*-test. (**C**) Number of differentially expressed genes (DEGs). The numbers of upregulated genes are indicated in red, and the numbers of downregulated genes are in blue. (**D**) Heat map showing the top functional pathways enriched in DEGs identified in (**C**). The heat map color intensity denotes the statistical significance of the enrichment (FDR at minus log 10 scale).

Additional file 1: Figure S3

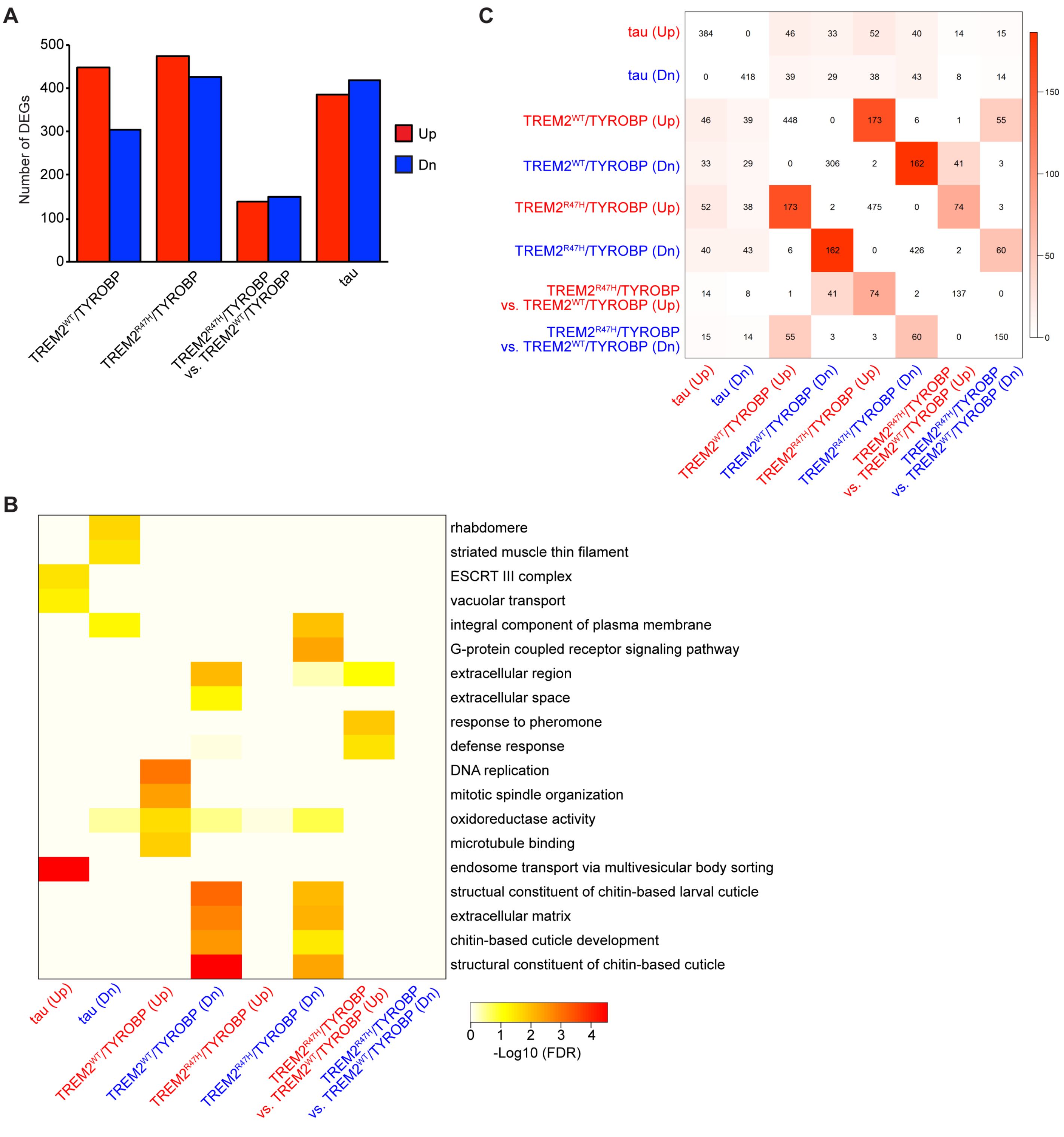

Figure S3. Molecular pathways affected by tau do not overlap with those affected by glial TREM2/TYROBP.

Additional file 1: Figure S3

(**A**) Number of differentially expressed genes (DEGs). The numbers of upregulated genes are indicated in red, and the numbers of downregulated genes are in blue. (**B**) Heat map showing the top functional pathways enriched in the DEGs identified in (**A**). The heat map color intensity denotes the statistical significance of the enrichment (FDR at minus log 10 scale). (**C**) Overlaps among DEGs identified in (**A**). The number in each cell indicates the number of common DEGs between row and column variables, with color intensity indicating the FDR adjusted P value at minus log 10 scale.

Additional file 1: Figure S4

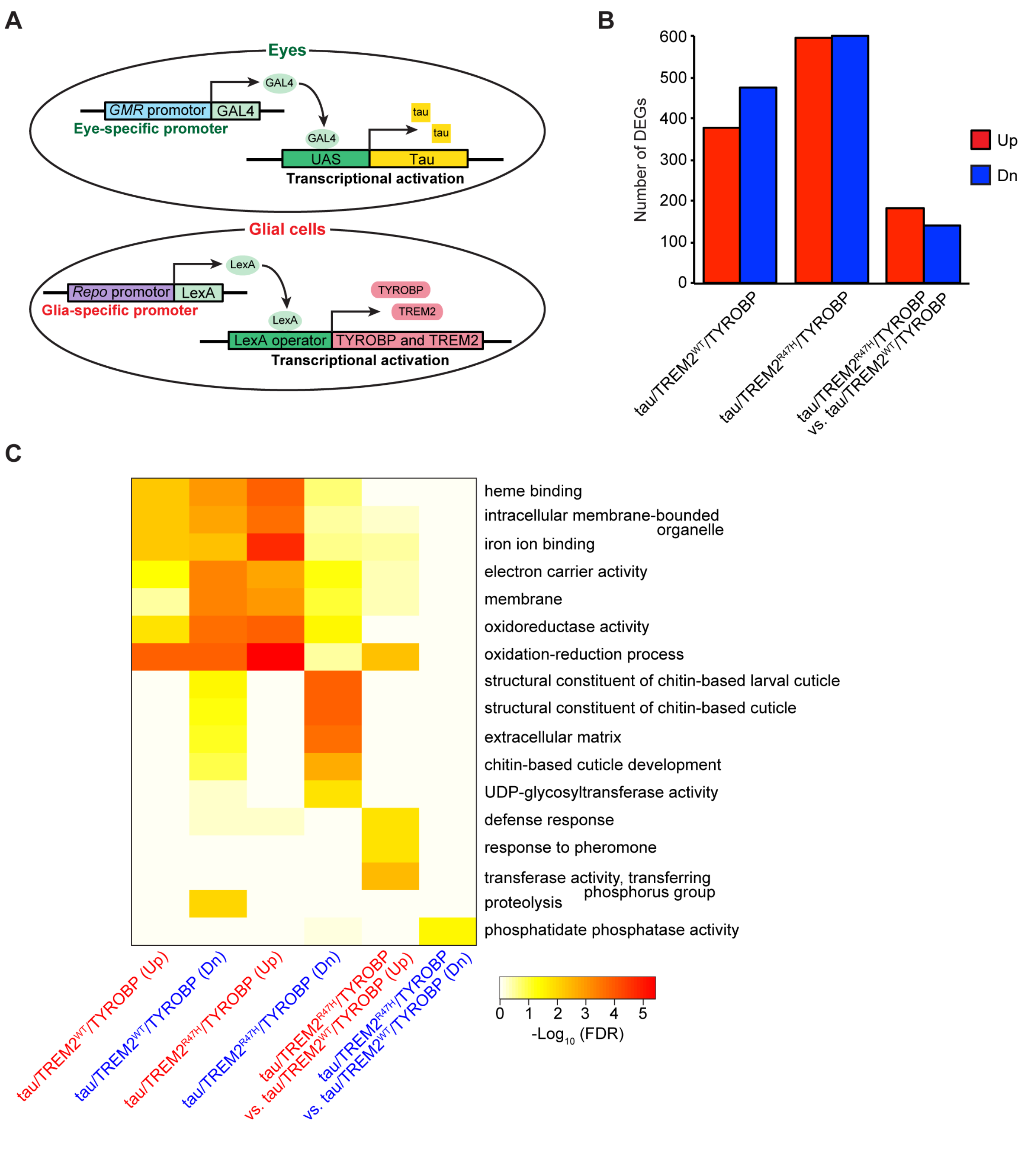

Figure S4. Gene expression signatures in tau/TREM2/TYROBP flies.

(**A**) Schematic representation of the transgene expression system of tau and TREM2/TYROBP in *Drosophila*. Human 0N4R tau was expressed in retina by GAL4-UAS system with pan-retinal GMR-GAL4 driver, while human TREM2/TYROBP complex was expressed in glial cells by LexA system with pan-glial Repo-LexA driver. (**B**) Number of differentially expressed genes (DEGs). The numbers of upregulated genes are indicated in red, and the numbers of down-regulated genes are in blue. (**C**) Heat map showing the top functional pathways

Additional file 1: Figure S4

enriched in DEGs identified in (**B**). The heat map color intensity denotes the statistical significance of the enrichment (FDR at minus log 10 scale).

Additional file 1: Figure S5

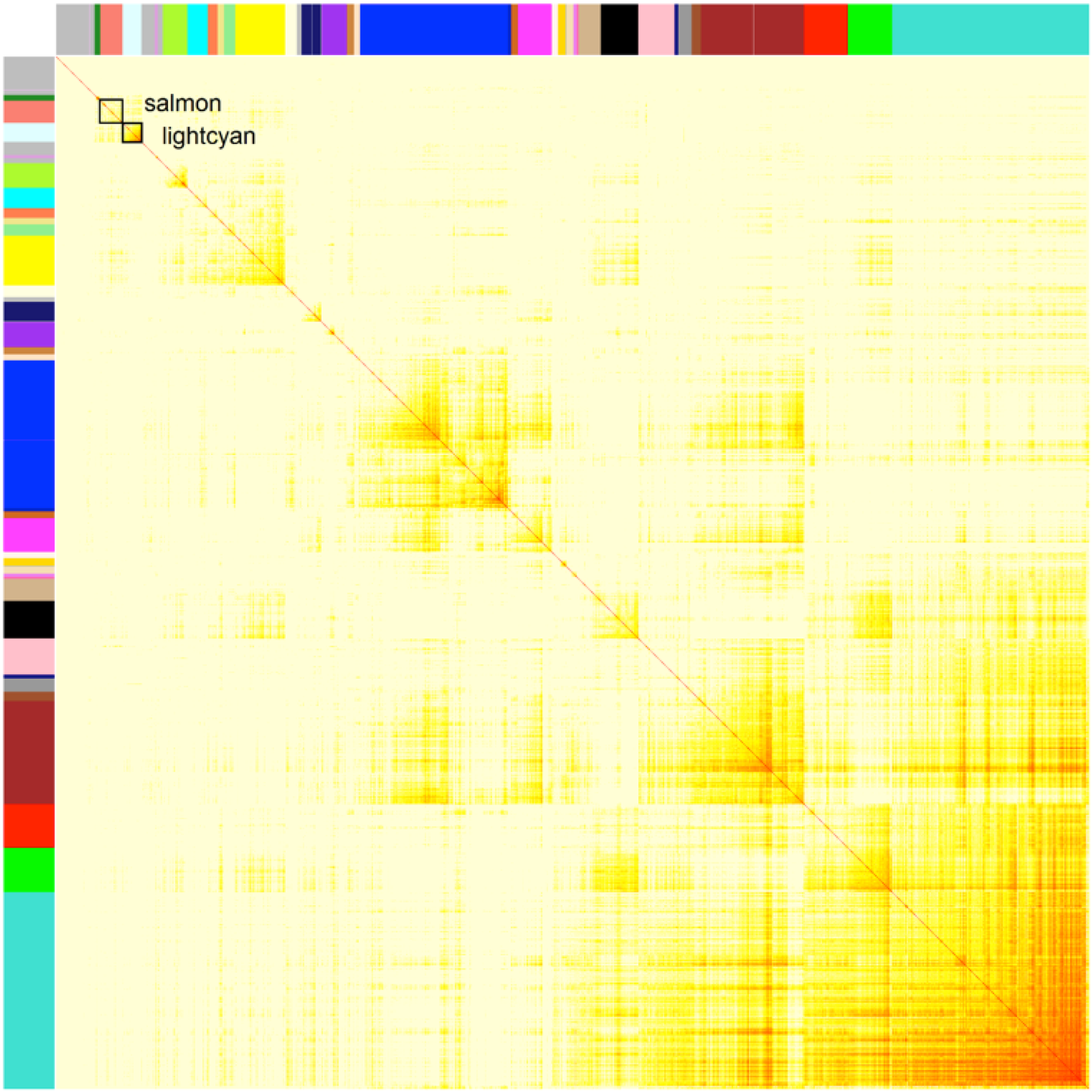

Figure S5. Heat-map showing the topological overlapping matrix (TOM) from weighted gene co-expression network analysis.

The color bars to the top and left of the heatmap show the module assignment. Two inflammatory response modules “salmon” and “lightcyan” are highlighted.

Additional file 1: Table S11

Table S11. Primer sequences for RT-PCR and qRT-PCR.

Name	Sequence	Product size
TREM2 (Human), Forward	5'- CTGCGGAATCTACAACCCCA -3'	164 bp
TREM2 (Human), Reverse	5'- TCGAAGCTCTCAGACTCCCC -3'	
TYROBP (Human), Forward	5'- AAGTGGTCTCCGTCCTGTCC -3'	199 bp
TYROBP (Human), Reverse	5'- AGTGATACGCTGTTTCCGGG -3'	
Sh (*Drosophila*), Forward	5'- TGTCAGGTTCCTCGCATGTC -3'	153 bp
Sh (*Drosophila*), Reverse	5'- CTGACTGGCGCTTTTGGAAG -3'	
SK (*Drosophila*), Forward	5'- GGTTATCGAAAACGAACTGAGCA -3'	135 bp
SK (*Drosophila*), Reverse	5'- ACTTCCAAAGCATGGTAAGCTAC-3'	
Shab (*Drosophila*), Forward	5'- CGTGCTCGCGTTTAGTGATG -3'	186 bp
Shab (*Drosophila*), Reverse	5'- TTCTGGTACTCGGCGCATTT -3'	
para (*Drosophila*), Forward	5'- ACGAGGATGAAGGTCCACAAC -3'	230 bp
para (*Drosophila*), Reverse	5'- ACGACGTATCGGATTGAATGG -3'	
Nmdar2 (*Drosophila*), Forward	5'- GGCATCCCGGTTATCTCGTG -3'	151 bp
Nmdar2 (*Drosophila*), Reverse	5'- AGAACTGGTGCCACTTGTAGC -3'	
